# Rougan Tongluo Decoction Initiates Neuroprotection Against Cerebral Ischemia by Activating the Endogenous SLC6A8‐Creatine‐EARS2 Mitochondrial Pathway

**DOI:** 10.1155/mi/4419137

**Published:** 2026-04-18

**Authors:** Changze Ou, Zheng-ping Bai, Guo-heng Hu, Binbin Chen, Da-hua Wu, Hua-jun Long

**Affiliations:** ^1^ Graduate School, Hunan University of Chinese Medicine, Changsha, 410208, Hunan, China, hnctcm.edu.cn; ^2^ Hunan Provincial Hospital of Integrated Traditional Chinese and Western Medicine (The Affiliated Hospital of Hunan Academy of Traditional Chinese Medicine), Changsha, 410006, Hunan, China; ^3^ The First Hospital of Hunan University of Chinese Medicine, Changsha, 410007, Hunan, China, hnctcm.edu.cn

**Keywords:** apoptosis, cerebral ischemic injury, creatine, EARS2, mitochondria, RGTL

## Abstract

**Objective:**

The traditional Chinese medicine formula Rougan Tongluo Decoction (RGTL) was widely used to treat neurological injury after cerebral ischemia, though its specific underlying mechanisms remain unknown. This study investigates the mechanisms via which RGTL helps alleviate cerebral ischemic injury to provide theoretical support for its application in cerebral ischemia treatment.

**Methods:**

A middle cerebral artery occlusion reperfusion (MCAO/R) rat model was established and treated with RGTL, N‐acetylcysteine (NAC), creatine, or sh‐EARS2. Network pharmacology and metabolomics were conducted to analyze the key efficacy‐related metabolites in the hippocampal tissue. An OGD/R cell model was constructed using PC12 cells and treated with creatine, RGTL‐containing serum, sh‐SLC6A8, and sh‐EARS2. Neuronal damage in the hippocampal tissues was assessed using HE and Nissl staining. Neuronal cell viability, mitochondrial membrane potential, and ROS levels were measured using CCK8, JC‐1, and DCFH‐DA assays. Mitochondrial damage was determined using transmission electron microscopy. The expression of SLC6A8/EARS2 axis and mitochondrial‐related proteins (cytochrome c [Cyt c]) was examined using RT‐qPCR and Western blot.

**Results:**

RGTL treatment reduced TNF‐α, IL‐6, and ROS levels while increasing ATP and JC‐1 in brain tissues of MCAO/R rats, thereby alleviating mitochondrial damage. The neuroprotective effects of RGTL were more pronounced than those of NAC. Succinic acid and creatine were identified as active drug ingredients and differential metabolites that may mediate RGTL’s therapeutic effects via MMP3, GAMT, SLC6A8, and CASP3. Silencing SLC6A8 abolished the protective effects of creatine against OGD/R‐induced neuronal cell apoptosis and mitochondrial damage. Creatine could bind to the EARS2 protein. Cell and animal experiments demonstrated that silencing EARS2 blocked the therapeutic effects of creatine in OGD/R and MCAO/R models, reversing its inhibition of neuronal apoptosis and mitochondrial damage.

**Conclusion:**

Creatine mediates the neuroprotective effects of RGTL by binding to EARS2, thus inhibiting mitochondrial damage and neuronal apoptosis to improve ischemic brain injury.

## 1. Introduction

Cerebral ischemia can arise due to disturbances in cerebral blood flow, which may damage brain tissues, causing neuronal cell death and cerebral infarction [1]. Cerebral artery embolism or thrombotic occlusion can trigger focal cerebral infarction, and delayed cerebral ischemia may occur due to blood vessel spasm after subarachnoid hemorrhage [1]. Endothelial cell injury can disrupt cerebral microcirculation, increase blood–brain barrier (BBB) permeability, and promote excessive production of reactive oxygen species (ROS), thereby leading to cerebral ischemic damage [2]. The Rougan Tongluo decoction (RGTL) is made using *Paeonia lactiflora*, *Salvia miltiorrhiza*, *Angelica sinensis*, *Pueraria lobata*, *Lycium barbarum*, *Morus alba*, *Eupolyphaga sinensis*, *Crataegus pinnatifida*, and *Polygonum multiflorum*. The formula is based on the pathological mechanisms of yin deficiency and blood stasis and was created according to the therapeutic principle of nourishing yin, promoting blood circulation, and unblocking collaterals. *Polygonum multiflorum*, *Lycium barbarum*, and *Morus alba* are known to nourish the liver and kidneys, and *Paeonia lactiflora*, *Salvia miltiorrhiza*, and *Angelica sinensis* have been shown to promote blood circulation and remove stasis. In addition, *Pueraria lobata* and *Eupolyphaga sinensis* can eliminate stasis and unblock colic laterals, and *Crataegus pinnatifida* may disperse stasis and transform phlegm. Together, these ingredients work synergistically to nourish yin, promote blood circulation, and unblock collaterals, thus addressing both the underlying cause and clinical manifestations, targeting not only the pathological mechanisms of ischemic wind, qi stagnation, and blood stasis but also the fundamental deficiency of the liver and kidneys associated with ischemic stroke. However, the mechanisms by which RGTL exerts its protective effects against cerebral ischemic injury remain unclear.

Creatine is also one of the medicinal ingredients of *Crataegus pinnatifida* in the RGTL decoction [3]. Studies have shown that creatine can be synthesized endogenously in the brain and cross the BBB to accumulate in the brain, although the uptake of creatine in the brain is typically limited [4]. A deficiency in brain creatine may lead to developmental delay, intellectual disabilities, and other neurological disorders. Creatine promotes the circulation of ATP, the energy source in brain tissue, and its neuroprotective effects against ischemic and oxidative damage are believed to result from the maintenance of mitochondrial membrane potential and the phosphocreatine system, which functions as an ATP buffer [5, 6]. Previous studies have also demonstrated that creatine exerts therapeutic effects in mouse models of ischemic stroke [7]. SLC6A8 mediates creatine transport into cells, and its high expression in organs with high demand for creatine, such as the brain and skeletal muscles, facilitates intracellular creatine accumulation along concentration gradients [8]. However, the role and mechanisms of creatine in cerebral ischemic injury remain to be further clarified.

Mitochondria are one of the earliest damaged organelles during ischemia and hypoxia [9], and mitochondrial dysfunction can lead to reduced ATP production, excessive ROS generation [10], as well as the release of mitochondrial DNA (mtDNA) into the cytoplasm [11]. Increased ROS production leads to oxidation of proteins and lipids, further impairing mitochondrial dynamics and inhibiting mitochondrial autophagy, ultimately aggravating mitochondrial dysfunction [12]. Glutamyl‐tRNA synthetase 2, encoded by *EARS2*, is a mitochondrial aminoacyl‐tRNA synthetase that plays an important role in mitochondrial translation [13]. Neural progenitor cells carrying *EARS2* mutations exhibit impaired neuronal development [14], and it is therefore speculated that creatine may alleviate mitochondrial damage and reduce neuronal apoptosis by modulating the EARS2 signaling pathway.

The DNA methyltransferase (DNMT) inhibitor RG108 has been shown to protect mitochondrial function by preventing ROS accumulation and inhibiting apoptosis [15], and maintaining mitochondrial function is essential for neuron survival and preserving nervous system function, as brain injury can cause acute mitochondrial damage and local energy failure, thereby accelerating neuronal death [16]. Damaged mitochondria promote neuronal apoptosis through the release of ROS and pro‐apoptotic factors [17]. Studies have reported that stabilization of L‐OPA1 can protect against ischemic brain injury by reducing neuronal apoptosis and preserving mitochondrial function [18]. Decreased levels of EARS2 protein have been shown to increase ROS production and alterations in mitochondrial morphology [19], while depletion of DARS2 in neurons induces mitochondrial dysfunction and neuronal cell apoptosis [20]. However, no studies have yet explored the role of EARS2 in cerebral ischemic injury, and it remains unclear whether creatine can regulate EARS2 expression. Thus, we speculated that creatine may upregulate EARS2 expression, suppress ROS production and mtDNA release, to alleviate mitochondrial damage and neuronal apoptosis.

In this study, we first proposed that RGTL might mediate brain tissue metabolism in rats with cerebral ischemic injury. Its active ingredient, creatine, was found to inhibit mitochondrial damage and neuronal cell apoptosis by regulating EARS2, leading to improvement in cerebral ischemic injury, thus providing insights into the potential pharmacological mechanism of RGTL in treating cerebral ischemic injury.

## 2. Methods

### 2.1. Drug Sources and Preparation

The RGTL decoction is composed of *Polygonum multiflorum* (15 g), *Morus alba* (15 g), *Lycium barbarum* (30 g), *Salvia miltiorrhiza* (30 g), *Pueraria lobata* (30 g), *Angelica sinensis* (10 g), *Paeonia lactiflora* (10 g), *Eupolyphaga sinensis* (10 g), and *Crataegus pinnatifida* (15 g). All herbs were supplied by the Affiliated Hospital of the Hunan Provincial Institute of Traditional Chinese Medicine and were authenticated as genuine Chinese medicinal materials. Briefly, the herbs were first boiled in 500 mL of water to obtain 150 mL of the initial decoction. A second decoction was then prepared by adding 300 mL of water to the residue, yielding another 150 mL, and the two decoctions were combined and simmered over low heat until a paste was formed, corresponding to a concentration equivalent to 2 g/mL of raw herbs. The decoction was cooled and stored in a refrigerator until use.

### 2.2. Network Pharmacology Analysis of RGTL and Cerebral Ischemic Injury

The compounds and targets of the ingredients of *Paeonia lactiflora*, *Salvia miltiorrhiza*, *Angelica sinensis*, *Pueraria lobata*, *Lycium barbarum*, and *Morus alba* were retrieved from the TCMSP database (https://tcmspw.com/tcmsp.php) using the screening criteria of oral bioavailability (OB) ≥ 30% and drug‐likeness (DL) ≥ 0.18 [21]. The compounds and targets of *Eupolyphaga sinensis*, *Crataegus pinnatifida*, and *Polygonum multiflorum* were obtained from the BATMAN‐TCM database (http://bionet.ncpsb.org.cn/batman-tcm/), and the components were selected based on a score > 0.99 [22]. All identified targets were standardized through the UniProt database (https://www.uniprot.org/), and non‐human targets were excluded [23].

Using “cerebral ischemic injury” as a keyword, human disease‐related genes were retrieved from the GeneCards database (https://www.genecards.org/) [24], NCBI Gene database (https://www.ncbi.nlm.nih.gov/) [25], and OMIM database (https://www.omim.org/) [26]. After deduplication, a total of 461 disease targets were obtained, and the drug targets and disease targets were entered into the Venny 2.1 software (https://bioinfogp.cnb.csic.es/tools/venny/index.html) to generate a Venn diagram, resulting in the identification of 127 common targets. These common drug‐disease targets were then imported into the STRING database (https://string-db.org/cgi/input.pl) to construct a protein–protein interaction (PPI) network, with the species set to “*Homo sapiens*” and the confidence level threshold set at > 0.4. Further, Gene Ontology (GO) and Kyoto Encyclopedia of Genes and Genomes (KEGG) pathway enrichment analyses were conducted using the STRING database, and entries with a corrected *p* < 0.05 were selected. The R software version 4.1.2 was used with the “clusterProfiler,” “enrichplot,” and “ggplot2” packages to generate bar plots of the enrichment results. By combining metabolomics findings with the identified key pharmacological components, succinic acid and creatine were obtained as representative active ingredients using Venny 2.1 software. The ingredient‐disease‐pathway‐target network file was subsequently imported into Cytoscape 3.8.0 (https://cytoscape.org/) to visualize the multi‐component, multi‐target interactions of the active ingredients, demonstrating their potential mechanisms in the treatment of cerebral ischemic injury.

### 2.3. Middle Cerebral Artery Occlusion Reperfusion (MCAO/R) Rat Intervention

Sprague‐Dawley (SD) rats (8 weeks old, 250–300 g) were fasted overnight and given free access to water. All experimental procedures were approved by the Animal Care and Use Committee of the Hunan Provincial Hospital of Integrated Traditional Chinese and Western Medicine (No. DWZX‐SB‐2024010) and conducted in accordance with the National Institutes of Health Guide for the Care and Use of Laboratory Animals. The next day, the rats were anesthetized by intraperitoneal injection of pentobarbital sodium (40 mg/kg) under sterile conditions. Each rat was placed in the supine position, and the neck skin was incised to expose the carotid artery. A 5 cm nylon suture was advanced through the middle carotid artery into the middle cerebral artery to a depth of 16–18 mm. After 2 h of occlusion, the filament was withdrawn to allow reperfusion, establishing the MCAO/R model. Both occlusion and reperfusion followed standardized criteria: cerebral blood flow was required to decrease to less than 20% of baseline during occlusion and to recover to more than 50% of baseline after filament withdrawal [27]. In the Sham group, the middle carotid artery was exposed but not ligated. The successfully modeled rats were randomly divided into 9 rats per group, and rats that failed reperfusion or died during modeling were excluded from the statistical analysis.

In experiment 1, the rats were randomly divided into Sham, MCAO/R, MCAO/R+Vehicle, and RGTL groups. Drug dosing was calculated according to the body surface area conversion method, using the daily human dose of RGTL decoction for a 70 kg adult (containing 165 g of crude herbs) as a reference. The decoction was diluted with distilled water and administered by gastric gavage at 1.4 mL/100 g body weight. A high dose of RGTL decoction was selected for this study, equivalent to 2.8 g/100 g in the RGTL group (~14.85 g/kg). The Sham and MCAO/R+Vehicle groups received equal volumes of physiological saline by gavage. RGTL was administered once daily for seven consecutive days before surgery. MCAO/R induction was performed 30 min after the final dose on day 7. Postoperatively, RGTL treatment was continued once daily for 3 days before rats were euthanized with sodium pentobarbital (50 mg/kg) for subsequent tissue collection.

In experiment 2, the rats were randomly divided into MCAO/R, RGTL, and N‐acetylcysteine (NAC) groups. The positive control drug NAC (50 mg/kg, Sigma, USA) was administered intraperitoneally before MCAO/R and for 3 days after MCAO/R [28, 29]. RGTL was administered by oral gavage on a matching schedule (30 min pre‐surgery and for 3 days postoperatively). The animals were euthanized after continuous administration for 3 days. The remaining groups will be handled in the same way.

In experiment 3, the rats were randomly divided into Sham, MCAO/R, Creatine, Creatine+sh‐NC, and Creatine+sh‐EARS2 groups. PC12 cells transfected with sh‐NC or sh‐EARS2 (5′‐AUAUGCAUGUAUGUGUUACAC‐3′, and 5′‐GUAACACAUACAUGCAUAUAU‐3′, HG‐LV001159493sh, Honorgene) lentivirus were then transplanted into the hippocampus of rats 24 h after MCAO/R [30, 31]. The anesthetized rats were fixed in a stereotaxic frame, the head was prepared, and disinfection was performed with a 75% alcohol swab. A 3 cm midline incision was made along the scalp, and the meningeal connective tissue was carefully dissected and removed from the skull surface to expose the anterior fontanel. A small hole (0.5 mm in diameter) was drilled at a site 2 mm posterior to the fontanel and 1.5 mm lateral to the sagittal suture. Approximately 10 μL of PBS containing about 1 × 10^10^ cells was slowly injected into the ipsilateral hemisphere (2–2.5 mm deep) of MCAO/R rats. The needle was withdrawn slowly, and the incision was sutured. Control rats received an equal volume of PBS. It is worth noting that, consistent with the RGTL dosing protocol, rats receiving exogenous creatine supplementation were administered 700 μL of sterile creatine solution (0.25 M) via daily intraperitoneal injection for a total of 10 days, while control rats received an equal volume of sterile PBS [32]. Specifically, creatine was administered once daily for seven consecutive days before MCAO/R induction, which was performed 30 min after the final creatine dose on day 7. Twenty‐four hours after MCAO/R, PC12 cells were transplanted intracerebrally, and creatine administration was continued for two additional days. Rats were euthanized 48 h after cell injection for brain tissue collection and subsequent analysis.

During the experiment, neurological function was assessed using the Longa scoring method based on the behavioral performance of the rats [33]. The scoring criteria were as follows: 0 points, no neurological deficit; 1 point, inability to fully extend the contralateral forelimb (left forepaw); 2 points, circling to the contralateral side; 3 points, leaning to the contralateral side; and 4 points, inability to move spontaneously accompanied by loss of consciousness.

### 2.4. Preparation of RGTL‐Containing Serum

After 7 days of oral administration of RGTL (8‐week‐old rats, ~250–300 g), the RGTL‐containing serum was prepared [34]. One hour after the final dose, rats were anesthetized with pentobarbital sodium by intraperitoneal injection, and blood was collected from the abdominal aorta under sterile conditions. The blood samples were centrifuged at 4°C (3000 r/min) for 15 min to obtain the supernatant, which was then inactivated in a 56°C water bath for 30 min and filtered through a 0.22 μm membrane for sterilization. The filtered serum was aliquoted and stored at −80°C for subsequent experiments.

### 2.5. Separation of Brain Tissue Mitochondria and 2,3,5‐Triphenyltetrazolium Chloride (TTC) Staining

After the experiment, the rats were euthanized, and their entire brain was quickly removed, following which the brain tissues were cut into coronal sections and stained with 2% TTC staining solution. After staining, the sections were washed with PBS and fixed in 10% neutral formaldehyde. The infarct area of each section was measured, and the total infarct volume was calculated.

The brain tissue was then suspended in mitochondrial isolation buffer (0.25 M sucrose, 10 mM Tris‐HCl, 1 mM EDTA, and 250 μg/mL bovine serum albumin, pH 7.4) and washed three times [35]. The brain tissue was then chopped with small scissors and manually homogenized with a glass homogenizer 10 times. The homogenate was centrifuged at 1000 × g for 10 min. The supernatant was collected and further centrifuged at 12,000 × g for 10 min. The mitochondrial pellet was washed once with isolation buffer and centrifuged at 12,000×g for 10 min. All procedures were conducted at 4°C.

### 2.6. Hematoxylin‐Eosin (HE) Staining

Brain tissue samples were fixed, sectioned, and baked at 60°C for 12 h. The sections were then immersed in xylene for 20 min, three times, followed by sequential immersion in 100%, 100%, 95%, 85%, and 75% ethanol, each for 5 min. After rehydration, the sections were rinsed with distilled water for 5 min. The sections were stained with hematoxylin (AWI0001a, Abiowell) for 1–10 min, rinsed with distilled water, and counterstained with PBS. They were then stained with eosin (AWI0029a, Abiowell) for 1–5 min and rinsed again with distilled water. Dehydration was performed through a graded ethanol series (95%–100%), each step lasting 5 min. Lastly, the sections were cleared twice in xylene for 10 min, mounted with neutral gum, and examined under a microscope (BA210T, Motic).

### 2.7. Nissl Staining

The sections were stained with Nissl’s stain (AWI0501a, Abiowell) for 0.5–1 min, depending on the desired staining intensity. Excess stain was washed off with distilled water. The sections were then differentiated with a 1% glacial acetic acid solution until the background appeared colorless under the microscope, with cells and Nissl bodies showing clear outlines. The sections were mounted with buffered glycerol and observed under a microscope.

### 2.8. Cell Experiment

The rat PC12 highly differentiated cells (AW‐CCR070, Abiowell) were cultured in RPMI‐1640, containing 10% fetal bovine serum, and 1% penicillin/streptomycin (1:1) (AW‐MC002, Abiowell). The cells were then cultured in a glucose‐free medium and then placed in an incubator at 37°C (0.5% O_2_, 5% CO_2_, and 94.5% N_2_) for 6 h. followed by culture in DMEM medium containing normal glucose [36]. Before the experiment, the cells were treated in a medium containing 1% FBS and 5 mM creatine (HY‐W010388, MCE) for 24 h [32]. The cells were treated with 0, 5, 10, and 20 mM creatine for OGD/R intervention [32].

To evaluate the transfection efficiency of SLC6A8 (HG‐RS017348, Honorgene), cells were divided into sh‐NC, sh‐SLC6A8#1 (5′‐AACAUUGAAGAUGAAGAUGCC‐3′, and 5′‐CAUCUUCAUCUUCAAUGUUGU‐3′), sh‐SLC6A8#2 (5′‐AAAUACCAGCGUAGUUGUGGG‐3′, and 5′‐CACAACUACGCUGGUAUUUUC‐3′), and sh‐SLC6A8#3 (5′‐UAAGAGAUAAGAGAUCUACAC‐3′, and 5′‐GUAGAUCUCUUAUCUCUUAGU‐3′) groups. To evaluate the effect of EARS2 silencing on creatine intervention in OGD/R cells, cells were divided into Control, OGD/R, Creatine, Creatine+sh‐NC, and Creatine+sh‐SLC6A8 groups.

To investigate the binding between creatine and EARS2 protein, the cells were divided into Control, Pronase+Creatine (5 μM), Pronase+Creatine (10 μM), and Pronase+Creatine (20 μM) groups, and Western blot was performed to detect the expression of EARS2 protein.

To evaluate the transfection efficiency of EARS2 (HG‐RS001159493, Honorgene), the cells were divided into Control, sh‐NC, sh‐EARS2#1 (5′‐UUCUGCUGCCCCCGGUACCAG‐3′, and 5′‐GGUACCGGGGGCAGCAGAAAA‐3′), sh‐EARS2#2 (5′‐UGUUACACAGGCAUACAUGGA‐3′, and 5′‐CAUGUAUGCCUGUGUAACACA‐3′), and sh‐EARS2#3 (5′‐AUAUGCAUGUAUGUGUUACAC‐3′, and 5′‐GUAACACAUACAUGCAUAUAU‐3′) groups. To investigate the effects of silencing EARS2 in creatine intervention on OGD/R cells, the cells were divided into Control, OGD/R, Creatine, Creatine+sh‐NC, and Creatine+sh‐EARS2 groups. To further explore the effects of silencing EARS2 in RGTL intervention on OGD/R cells, OGD/R cells were incubated for 24 h with normal serum or serum containing 0%, 5%, 15%, or 20% medication [34]. The cells were divided into Control, OGD/R, Normal serum (without medication), medication‐containing (RGTL) serum groups, RGTL serum+sh‐NC group, and RGTL serum+sh‐EARS2 groups.

### 2.9. CCK8

Cells from the above groups were seeded into 96‐well plates at a density of 5 × 10^3^ cells per well, with 100 μL per well. Then, 10 μL of CCK8 reagent was added to each well, and the plate was incubated at 37°C with 5% CO_2_ for 4 h [37]. Absorbance at 450 nm was measured using a microplate reader, and the mean values were used to generate a bar graph.

### 2.10. JC‐1 Assay

The cells were suspended in 0.5 mL of cell culture medium containing serum and phenol red. Then, 0.5 mL of JC‐1 staining working solution (C2006, Beyotime) was added and gently mixed by inversion. The cells were incubated at 37°C for 20 min in a cell culture incubator. After incubation, the cells were centrifuged at 600×g at 4°C for 5 min to collect the pellet. The pellet was resuspended in 1 mL of JC‐1 staining buffer (1×), centrifuged again under the same conditions, and the supernatant was discarded. This washing step was repeated once more with 1 mL of JC‐1 staining buffer (1×). Finally, the cells were resuspended in JC‐1 staining buffer (1×) and analyzed using a flow cytometer (A00‐1‐1102, Beckman).

### 2.11. ROS Assay

The treated cells were removed from the culture medium, and an appropriate volume of diluted DCFH‐DA solution (S0033S, Beyotime) was added. The cells were incubated at 37°C in a cell culture incubator for 20 min. After incubation, they were washed three times with serum‐free culture medium to completely remove any DCFH‐DA that had not entered the cells. The cells were then collected by trypsin digestion and analyzed using a flow cytometer.

### 2.12. RT‐qPCR

Total RNA from cells or tissues was extracted using TRIzol reagent (15596026, Thermo, USA). cDNA was synthesized using an mRNA reverse transcription kit (CW2569, Kangwei Century, Beijing, China). The expression levels of target genes were quantified using the UltraSYBR Mixture kit (CW2601, Kangwei Century, Beijing, China) on a fluorescence quantitative PCR instrument (PIKOREAL96, Thermo, USA). Gene expression was calculated using the 2^−ΔΔCt^ method (Table 1).

**Table 1:** Primer sequence.

Gene	Sequence (5′‐3′)	Length (bp)
M‐β‐Globin	F: CCACCTGGGCAAGGATTTCA	129
	R: AACCATTGTTCACAGGCAAGA	
M‐mt‐ND1	F: ATCAGGATGAGCCTCAAACTCC	187
	R: TACTTCTGCCAGCCTGACCC	
M‐SLC6A8	F: CTCCCGGCCTCCTACTACTT	106
	R: ACATTCCACCATCAGTCACCAT	
M‐actin	F: ACATCCGTAAAGACCTCTATGCC	223
	R: TACTCCTGCTTGCTGATCCAC	

### 2.13. Western Blot

After the experiment, tissue and cell samples were collected and lysed using Radioimmunoprecipitation Assay (RIPA) (AWB0136, Abiowell) and lysis buffer. Protein concentrations were determined using the Bicinchoninic Acid (BCA) method. Equal amounts of protein were separated by sodium dodecyl sulfate‐polyacrylamide gel electrophoresis (SDS‐PAGE) and transferred onto polyvinylidene difluoride (PVDF) membranes pre‐activated with methanol. The membranes were blocked with 5% skim milk and dried for at least 1 h at room temperature. The membranes were incubated overnight at 4°C with primary antibodies, followed by incubation with HRP‐conjugated secondary anti‐IgG antibodies (SA00001‐1/2, 1:5000/6000, Proteintech, USA) for 90 min at 37°C (Table 2). Protein bands were visualized using a chemiluminescence detection kit (AWB0005, Abiowell), and image analysis was performed using ChemiScope 6100 software (Qingxiang, China).

**Table 2:** Antibody information.

Proteins	No.	Source	Ratio	Weight (kDa)	Time (min)	Company
SLC6A8	20299‐1‐AP	Rabbit	1:500	65–70	90	Abcam
Bax	ab32503	Rabbit	1:5000	21	40	Abcam
Bcl2	ab182858	Rabbit	1:2000	26	50	Abcam
Caspase3	19677‐1‐AP	Rabbit	1:1000	17, 19, 32–35	60	Proteintech
Cytochrome c	AWA10373	Rabbit	1:2000	12	40	Abiowell
EARS2	PA5‐113954	Rabbit	1:1000	50	70	Thermofisher
β‐actin	AWA80002	Rabbit	1:5000	42	60	Abiowell
COX IV	ab33985	Mouse	1:1000	15	40	Abcam

### 2.14. Transmission Electron Microscopy

Cells and tissue sections were pre‐fixed with glutaraldehyde (AWI0097, Abiowell) and subsequently further fixed with 1% osmium tetroxide. The sections were dehydrated stepwise with acetone (10000418, Xilong Scientific Co., Ltd.) using a graded concentration series of 30%, 50%, 70%, 80%, 90%, 95%, and 100%. After dehydration, the sections were infiltrated with mixtures of dehydration agent and Epon‐812 embedding resin at ratios of 3:1, 1:1, and 1:3, followed by embedding in pure Epon‐812 resin (GS02659, Beijing Zhongjing Ke Instrument Technology Co., Ltd.). Ultrathin sections (60–90 nm) were cut using an ultramicrotome and mounted onto copper grids (BZ11262a, Beijing Zhongjing Ke Instrument Technology Co., Ltd.). The grids were stained with uranyl acetate for 10–15 min and then with lead citrate (GA10701‐1, Beijing Zhongjing Ke Instrument Technology Co., Ltd.) for 1–2 min at room temperature. Imaging was performed using a JEM‐1400FLASH transmission electron microscope (JEOL).

### 2.15. Molecular Docking

Creatine (https://pubchem.ncbi.nlm.nih.gov/#query=creatine&tab=compound) and the EARS2 protein (https://www.uniprot.org/uniprotkb?query=EARS2) were docked using the VINA 1.1.2 software, which applies a semi‐empirical free energy force field to predict the binding energies between receptor and ligands. Structural analysis was performed using PYMOL to confirm whether the compounds could stably bind to the protein cavity and interact with the surrounding amino acids.

### 2.16. Metabolomics

The tissues were transferred to a −4°C refrigerator for thawing. Then, ~150 mg of tissue was weighed using a microbalance and placed into a sterile Eppendorf tube for cutting. Subsequently, 300 μL of double‐distilled water and 600 μL of methanol were added to the tissue, and the sample was homogenized until no visible tissue fragments remained using a homogenizer. Next, 300 μL of chloroform was added, and all the above procedures were performed on ice. The mixture was then subjected to 5 min of ultrasound treatment, followed by centrifugation in a refrigerated centrifuge (−4°C, 13,000 rpm, 15 min). The resulting supernatant (850 μL) was transferred to another sterile Eppendorf tube, and concentrated to dryness by nitrogen blowing in a concentrator to completely remove methanol. Before NMR analysis, 600 μL of D_2_O containing 0.015% TSP was added to the samples, followed by 10 min of sonication to mix them thoroughly. Subsequently, the mixture was centrifuged at −4°C, 13,000 rpm for 15 min, and 500 μL of the clear solution was transferred to a 5 mm NMR tube, sealed with a rubber cap, and analyzed using a Bruker 600 MHz spectrometer for ^1^H ‐NMR detection. The NOESY‐PR‐1D pulse sequence was applied with the following parameters: spectral width, 12.019 kHz; relaxation time, 320 ms; number of scans, 64; FID transformation, LB = 0.3 Hz; PW = 30°C (12.7 μs); and RD = 1.0 s. After acquiring the metabolite spectra, all ^1^H‐NMR data were processed for denoising, baseline correction, and phase correction using the MestReNova software (MestReNova v9.0.1). The spectra were aligned using the TSP peak at 0.00 ppm as a reference, and the water region (4.75–4.85 ppm) was removed. The spectral region from *δ* 0.6 to 9.5 ppm was segmented at intervals of 0.01 and integrated, followed by normalization of the integration values to compensate for differences in sample concentrations. Next, the normalized integration values were imported into SIMCA‐P software for analysis by unsupervised principal component analysis (PCA) and supervised orthogonal partial least squares discriminant analysis (OPLS‐DA). Differential metabolites were identified by combining results from an independent sample *t*‐test (*p*‐value <0.05) and substances with VIP ≥ 1 in the S‐plots of the OPLS‐DA model. Finally, metabolic pathway analysis was performed using the online platform MetaboAnalyst (https://www.metaboanalyst.ca/).

### 2.17. ELISA

According to the manufacturers’ instructions, ATP (A095‐1‐1, njjcbio), IL‐6 (CSB‐E04640r, cusabio), and TNF‐α (CSB‐E11987r, Cusabio) kits were used to detect the levels of ATP, IL‐6, and TNF‐α in cell supernatants or tissue homogenate supernatants, respectively.

### 2.18. Small Molecule Pull‐Down

A small molecule pull‐down experiment was employed to detect the binding of creatine to the EARS2 protein. In brief, after biotin labeling, creatine was coupled with streptavidin magnetic beads (HY‐K0208, MCE). The coupled magnetic beads were incubated with the cell lysate at room temperature for 60 min, washed with PBS to remove unbound proteins. Then, 5× SDS‐PAGE protein loading buffer was added, the mixture was boiled for 5 min, and the supernatant was removed to obtain the pull‐down samples for Western blot analysis with EARS2 (PA5‐113954, 1:1000, Thermofisher). Meanwhile, the total protein (input) was set as a reference.

### 2.19. Statistical Analysis

Data analysis was conducted using Graphpad Prism 8.0 statistical software. Measurement data are presented as mean ± standard deviation. Normality and homogeneity of variance tests were conducted first. When the data met the assumptions of normal distribution and homogeneity of variance, intergroup comparisons were analyzed using an unpaired *t*‐test. For comparisons among multiple groups, one‐way analysis of variance (ANOVA) or repeated measures ANOVA was performed, followed by Tukey’s post hoc test. *P* < 0.05 was used to indicate statistically significant differences.

## 3. Results

### 3.1. RGTL Improves Mitochondrial and Neuronal Damage Related to Cerebral Ischemia

MCAO/R rats showed an increase in the neurological deficit score, but it was reduced after the RGTL treatment (Figure 1A). TTC staining revealed an increase in the infarct area of MCAO/R rat brain tissue, but a decrease in the infarct area in the RGTL treatment group (Figure 1B). Pathological observations showed significant shrinkage of hippocampal neurons in MCAO/R rats, with smaller volume and darker nuclear staining (Figure 1C). In the RGTL group, hippocampal lesions decreased, with normal cell morphology and structure, occasional reduction in cell volume, and lighter nuclear staining (Figure 1C). Nissl staining showed ruptured hippocampal neurons in MCAO/R rats, irregular arrangement, and incomplete cell bodies, while RGTL treatment alleviated these changes (Figure 1D). Further analysis revealed increased levels of TNF‐α, IL‐6, and ROS in the hippocampal tissue of MCAO/R rats, along with decreased ATP levels (Figure 1E, F, Supporting information Figure S1A). RGTL treatment reduced TNF‐α, IL‐6, and ROS levels in hippocampal tissue and increased ATP levels (Figure 1E, F, Supporting information Figure S1A). Mitochondrial membrane potential detection showed a decrease in mitochondrial membrane potential in the hippocampal tissue of MCAO/R rats, which increased after RGTL treatment (Figure 1G, Supporting information Figure S1B). Transmission electron microscopy further revealed mitochondrial fragmentation and vacuolization in the brain tissue of MCAO/R rats, while RGTL treatment alleviated mitochondrial damage (Figure 1H). These results indicate that RGTL treatment effectively improved mitochondrial and neuronal damage, as well as neurological function, in MCAO/R rats.

Figure 1RGTL improves mitochondrial and neuronal damage in cerebral ischemia rats. (A) Neurological function deficit score. (B) TTC staining detects cerebral infarction in rat brain tissues. (C) HE staining to assess brain tissue damage in rats (Scale bar = 100 μm or 25 μm). (D) Nissl staining to observe damage in rat hippocampal neurons (Scale bar = 100 μm or 25 μm). (E) ELISA to measure levels of inflammatory factors TNF‐α, IL‐6, and ATP content. (F) DCFH‐DA staining to detect ROS levels. (G) Flow cytometry to measure mitochondrial membrane potential. (H) Transmission electron microscopy to observe brain tissue mitochondrial damage (Scale bar = 2.0 μm and 500 nm). MCAO/R rats were treated with either RGTL (MCAO/R+RGTL group) or saline (MCAO/R+Vehicle group) to evaluate the therapeutic effect. *N* = 9 mice/group.

**Figure:**
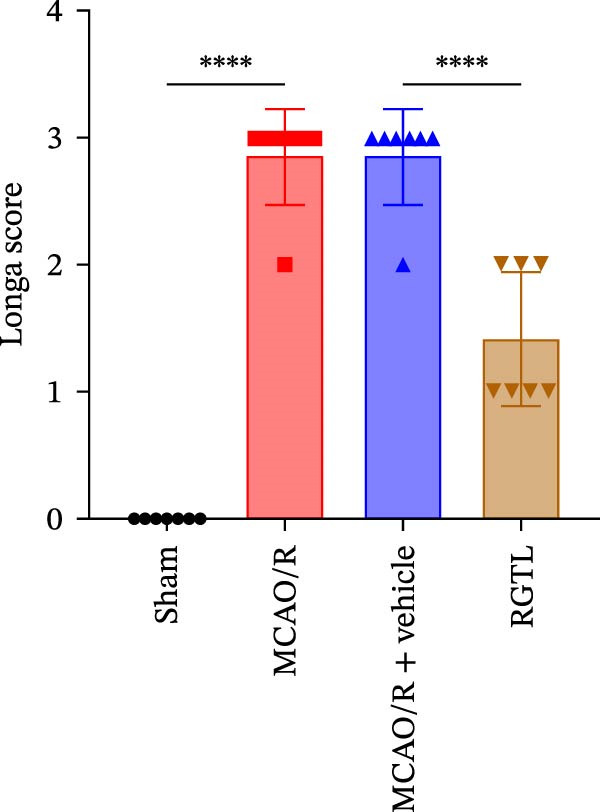
(A)

**Figure:**
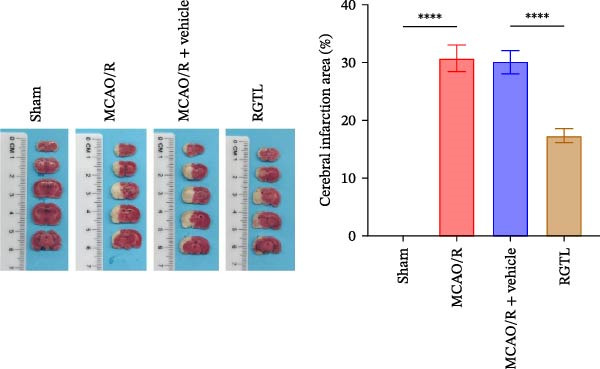
(B)

**Figure:**
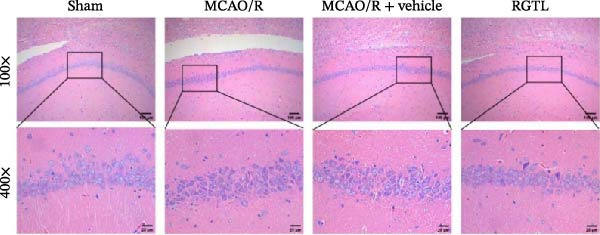
(C)

**Figure:**
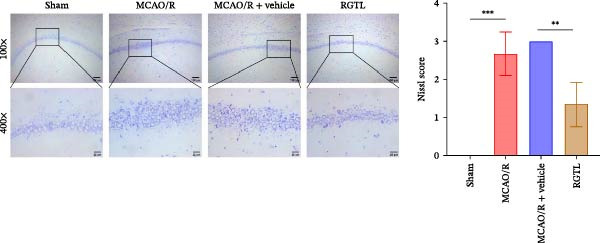
(D)

**Figure:**
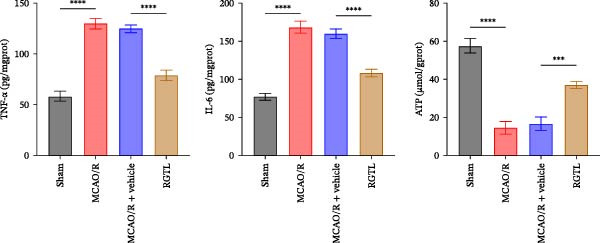
(E)

**Figure:**
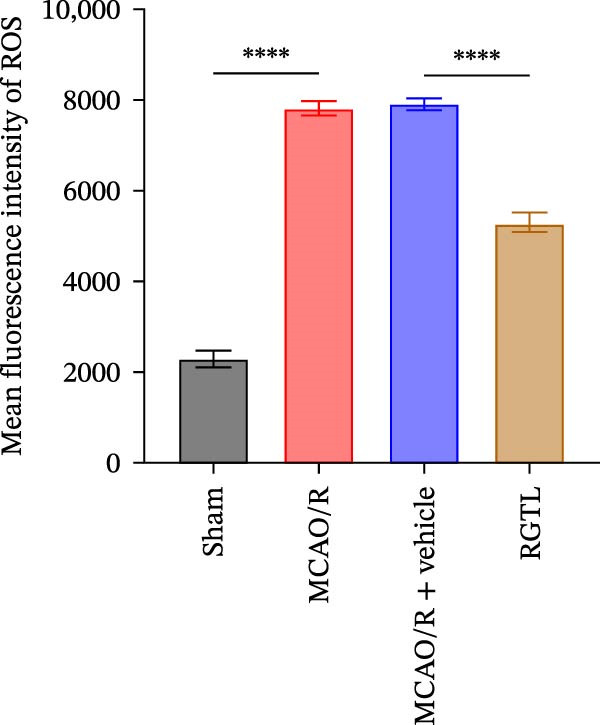
(F)

**Figure:**
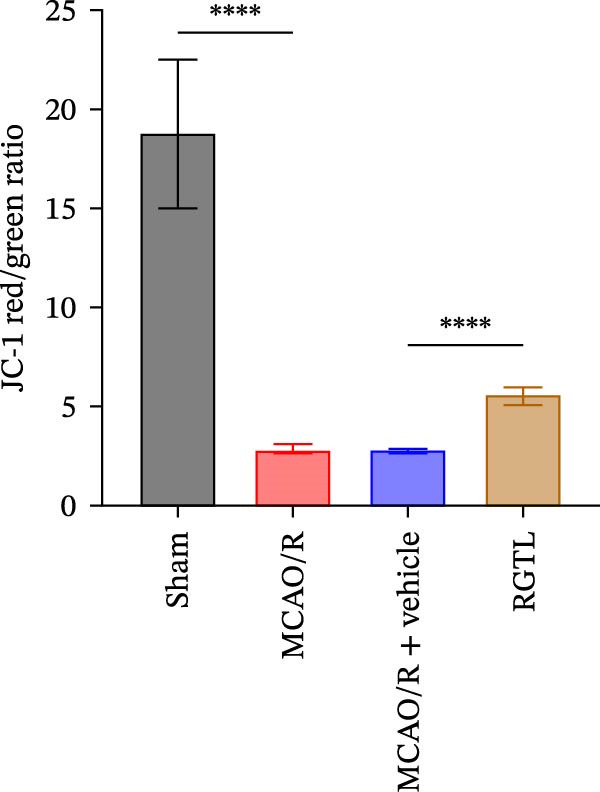
(G)

**Figure:**
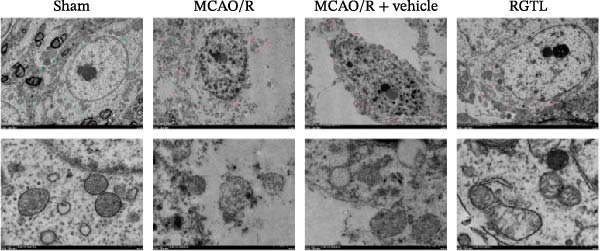
(H)

To evaluate the therapeutic effect of RGTL, we conducted an animal experiment using NAC as the positive control. MCAO/R rats showed an increase in the neurological deficit score, but it was reduced after the RGTL and NAC treatment (Supporting information Figure S2A). TTC staining revealed an increase in the infarct area of MCAO/R rat brain tissue, but a decrease in the infarct area in the RGTL and NAC treatment groups (Supporting information Figure S2B). Pathological observations showed significant shrinkage of hippocampal neurons in MCAO/R rats, with smaller volume and darker nuclear staining (Supporting information Figure S2C). In the RGTL and NAC groups, hippocampal lesions decreased, with normal cell morphology and structure, occasional reduction in cell volume, and lighter nuclear staining (Supporting information Figure S2C). Nissl staining showed ruptured hippocampal neurons in MCAO/R rats, irregular arrangement, and incomplete cell bodies, while RGTL and NAC treatment alleviated these changes (Supporting information Figure S2D). The neuroprotective effects of RGTL are consistent with the treatment of NAC.

### 3.2. Metabolomics Analysis of Differential Metabolites in the Brain of Rats With Cerebral Ischemic Injury Treated With RGTL

The changes in metabolic characteristics of brain tissue in MCAO/R rats after RGTL treatment were further analyzed using metabolomics. PCA analysis showed a clear separation between the sham and MCAO/R groups, indicating altered brain metabolism in MCAO/R rats (Figure 2A). The RGTL treatment group was positioned between the sham and MCAO/R groups, suggesting that RGTL partially restored the metabolic profile of brain tissue in MCAO/R rats (Figure 2A). The heatmap further illustrated the changes in the abundance of differential metabolites in MCAO/R rats before and after RGTL treatment (Figure 2B). These findings indicate that RGTL treatment improved the metabolic characteristics of brain tissue in MCAO/R rats, making them more similar to those of the sham group.

Figure 2Metabolomics analysis confirms that RGTL improves brain metabolic features in rats with cerebral ischemic injury. (A) PCA plot showing differences in metabolic features between groups. (B) Heatmap showing the abundance of differential metabolites. MCAO/R rats were treated with RGTL to analyze the metabolism of brain tissue. *N* = 3 biological repetitions/group.

**Figure:**
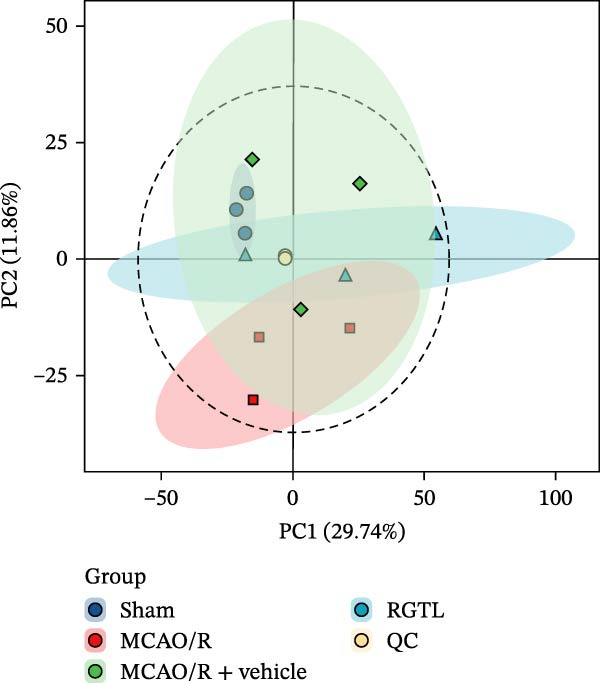
(A)

**Figure:**
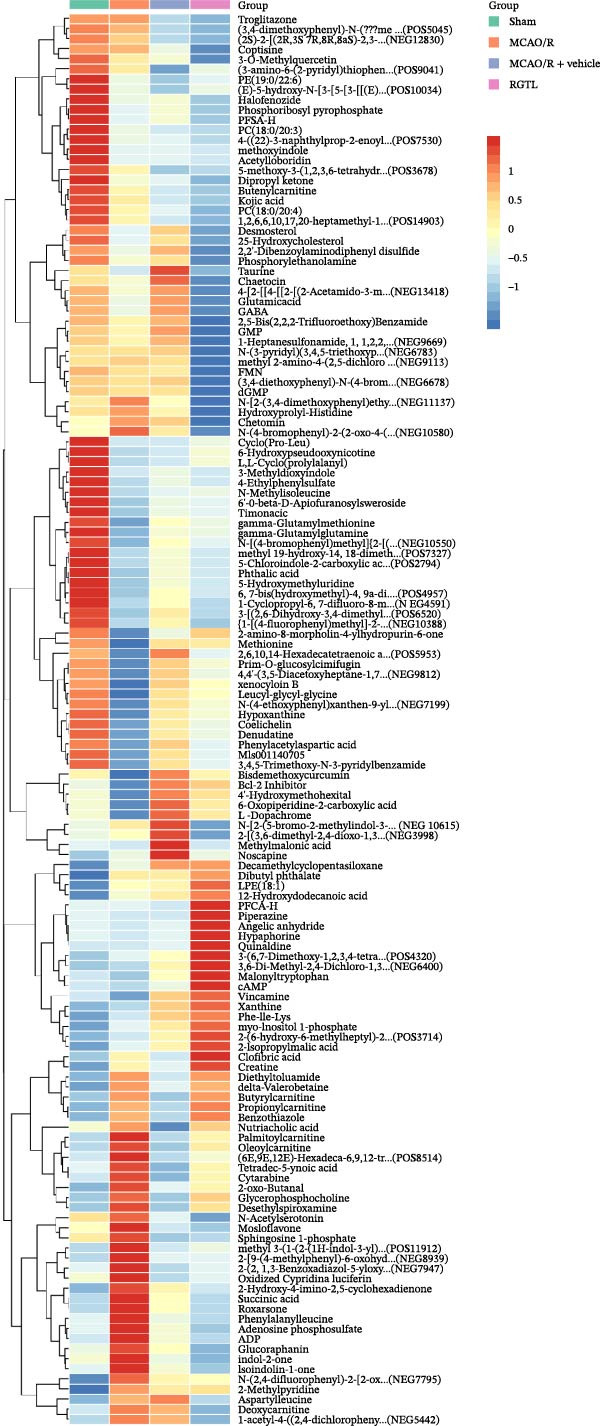
(B)

### 3.3. Comprehensive Analysis of Metabolomics and Network Pharmacology to Obtain Key Differential Metabolites Succinic Acid and Creatine

Further network pharmacology analysis showed that RGTL alleviates cerebral ischemic injury through a shared gene interaction network (Figure 3A). Functional enrichment analysis revealed that the reactive oxygen species metabolic process, membrane raft, and signaling receptor activator activity pathways were significantly enriched in the GO database, while the lipid and atherosclerosis pathways were significantly enriched in the KEGG database (Figure 3B). The Venn diagram demonstrated the intersection of differential metabolites and drug components, identifying succinic acid and creatine as shared components (Figure 3C). Further construction of the interaction network between drug components and target genes indicated that succinic acid is involved in ischemia/reperfusion injury through MMP3 and CASP3, while creatine is involved in I/R through GAMT, SLC6A8, and CASP3 (Figure 3D). These results confirm that succinic acid and creatine, as active components and differential metabolites in hippocampal tissue, may mediate the therapeutic effects of RGTL in MCAO/R rats via MMP3, GAMT, SLC6A8, and CASP3.

Figure 3Succinic acid and creatine are identified as key pharmacodynamic components in RGTL treatment of MCAO/R. (A) PPI network diagram of RGTL and I/R common genes. (B) GO and KEGG enrichment analyses of common genes. (C) Venn diagram showing the intersection of differential metabolites and drug components. (D) I/R‐pathway‐target network diagram of succinic acid and creatine. *N* = 3 biological repetitions/group.

**Figure:**
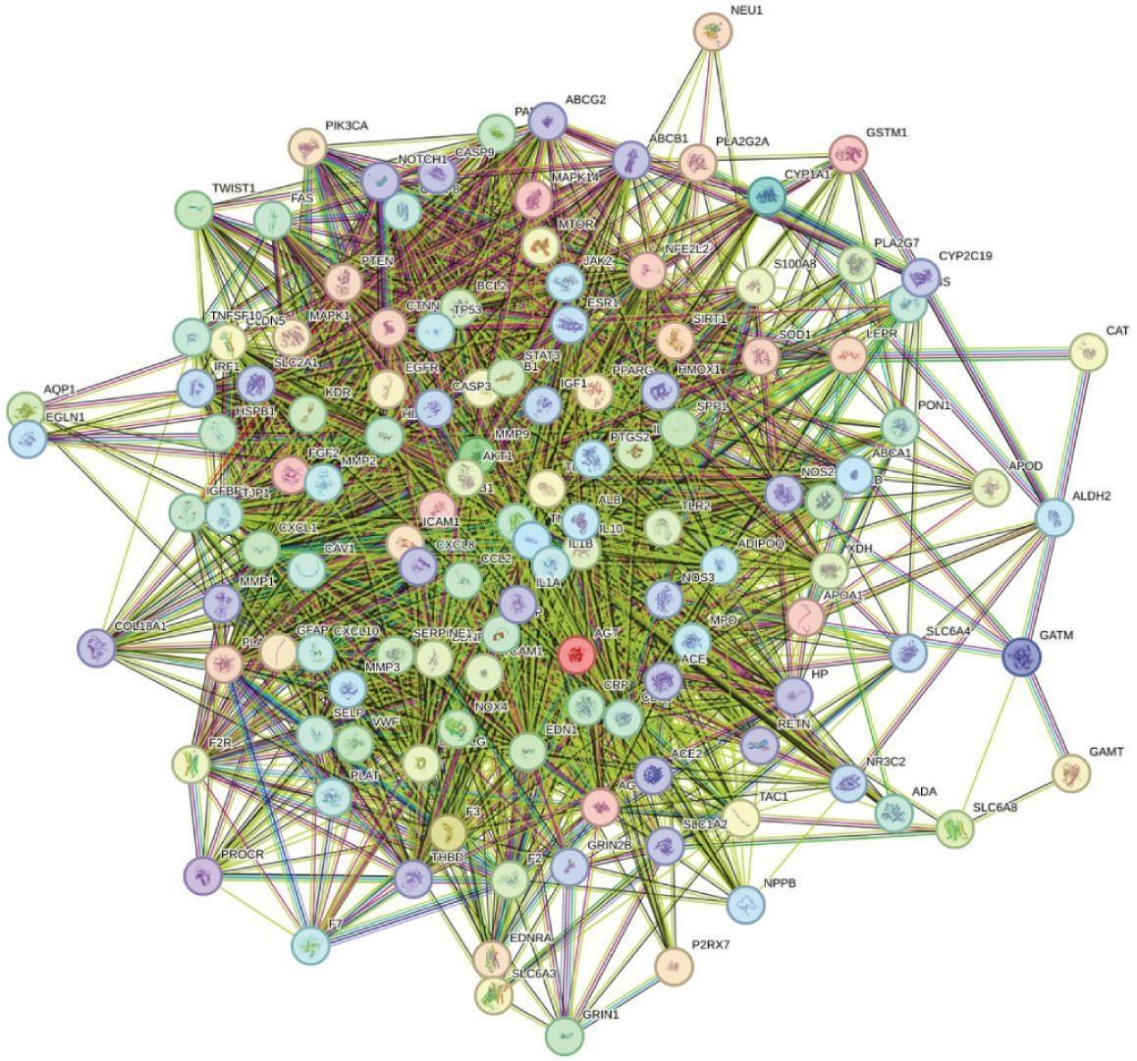
(A)

**Figure:**
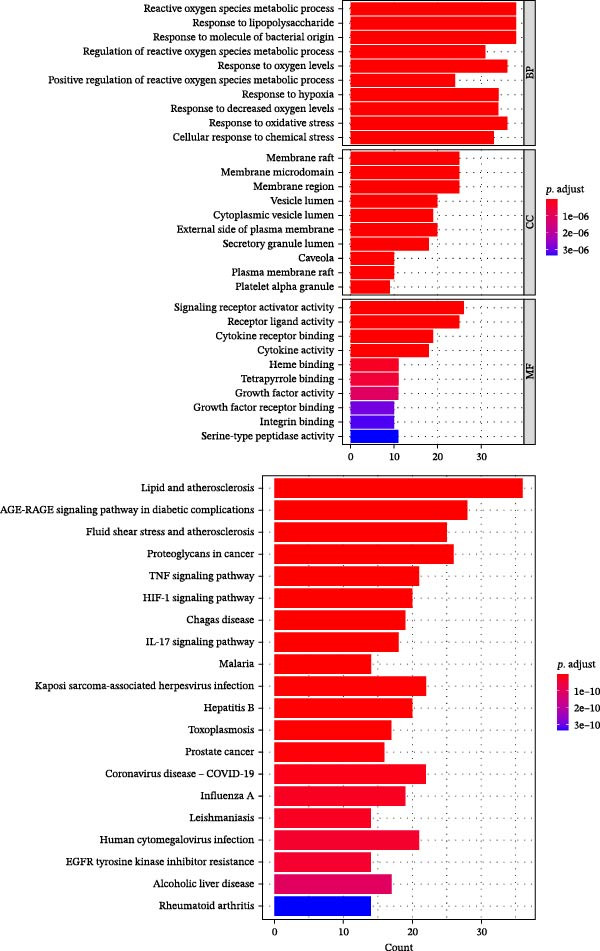
(B)

**Figure:**
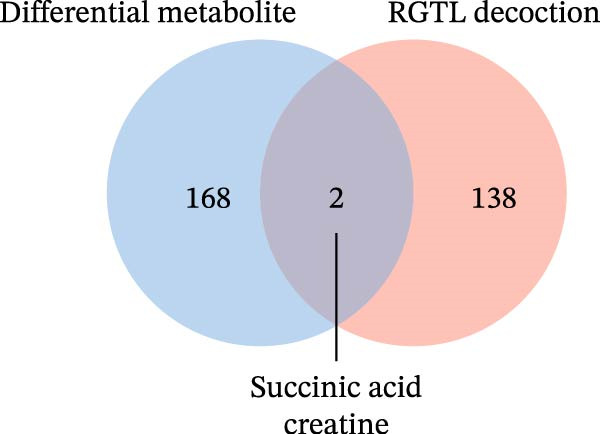
(C)

**Figure:**
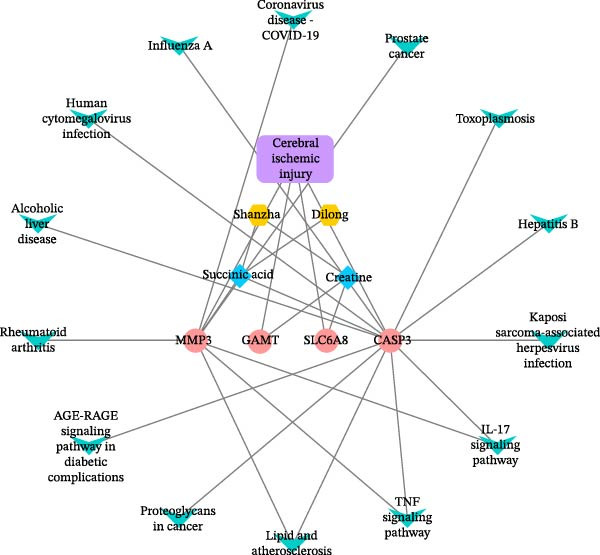
(D)

### 3.4. Creatine Relies on SLC6A8 to Inhibit Mitochondrial Damage and Neuronal Apoptosis

Previous studies have shown that although AGAT, GAMT, and SLC6A8 are expressed in the central nervous system (CNS) of mammals, the absence of SLC6A8 in astrocytes surrounding the BBB limits the brain’s ability to uptake creatine from the periphery [38], and the CNS primarily depends on AGAT and GAMT expression for endogenous creatine synthesis. Based on this, SLC6A8 was selected as the target to further explore the potential mechanisms of the hippocampal tissue metabolite creatine. Neuronal cells were first transfected with sh‐SLC6A8 for subsequent analysis (Figure 4A). Neuronal cells subjected to OGD/R were then treated with 0, 5, 10, and 20 mM creatine. The results showed that 5, 10, and 20 mM creatine all restored cell viability (Figure 4B). However, compared with the 10 mM group, 20 mM creatine showed no significant difference (Figure 4B). Therefore, 10 mM creatine was used for subsequent experiments. Compared with the Control group, SLC6A8 expression increased in OGD/R‐induced neuronal cells (Figure 4C). Creatine treatment had no significant effect on SLC6A8 expression in OGD/R‐induced neuronal cells, but simultaneous sh‐SLC6A8 intervention inhibited SLC6A8 expression (Figure 4C). Furthermore, creatine treatment improved the reduced viability of OGD/R‐induced neuronal cells, but this effect was blocked by sh‐SLC6A8 intervention (Figure 4D). ROS levels increased, and ATP levels decreased in OGD/R‐induced neuronal cells, both of which were reversed after creatine treatment (Figure 4E, F, Supporting information Figure S1C). When sh‐SLC6A8 was introduced, ROS levels increased, and ATP levels decreased again (Figure 4E, F, Supprting information Figure S1C). Known mtDNA damage and cytochrome c (Cyt c) release are key indicators of mitochondrial dysfunction [39]. Further analysis revealed that OGD/R induction reduced mitochondrial mtDNA copy number, mitochondrial Cyt c (Mito‐Cyt c), and JC‐1 levels in neuronal cells, while cytosolic Cyt c (Cyto‐Cyt c) levels increased (Figure 4G–I, Supporting information Figure S1D). Creatine treatment prevented the decrease in mtDNA copy number, Mito‐Cyt c, and JC‐1 levels, and the increase in Cyto‐Cyt c levels; however, this protective effect was abolished by sh‐SLC6A8 intervention (Figure 4G–I, Supporting information Figure S1D). In addition, creatine treatment suppressed the reduction in Bcl‐2 expression and the increase in BAX and caspase‐3 expression in OGD/R‐induced neuronal cells, while this effect was reversed by sh‐SLC6A8 intervention (Figure 4J). These results confirm that silencing SLC6A8 blocks the therapeutic effects of creatine on apoptosis and mitochondrial damage induced by OGD/R in neuronal cells.

Figure 4Creatine alleviates OGD/R‐induced neuronal cell apoptosis and mitochondrial damage through SLC6A8. (A) RT‐qPCR and WB detection of knockdown efficiency of SLC6A8. (B) CCK8 detection of neuronal cell viability. (C) RT‐qPCR and WB detection of SLC6A8 expression in cells. (D) CCK8 detection of neuronal cell viability. (E) DCFH‐DA staining to detect ROS levels. (F) Measurement of ATP levels using a commercial kit. (G) RT‐qPCR detection of mitochondrial mtDNA copy number. (H) WB detection of mitochondrial cytochrome c and cytoplasmic cytochrome c levels. (I) JC‐1 detection of mitochondrial membrane potential. (J) WB detection of expression of Bcl‐2, BAX, and caspase‐3. An OGD/R cell model was constructed in vitro with PC12 cells, and treated with creatine, and transfected with sh‐NC/sh‐SLC6A8. *N* = 3 biological repetitions/group.

**Figure:**
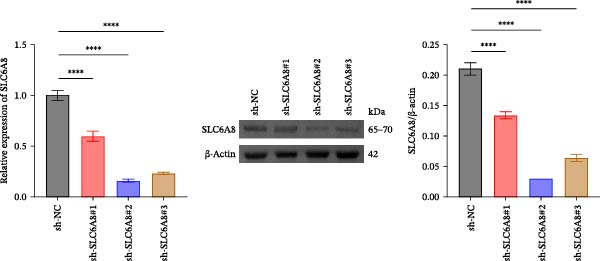
(A)

**Figure:**
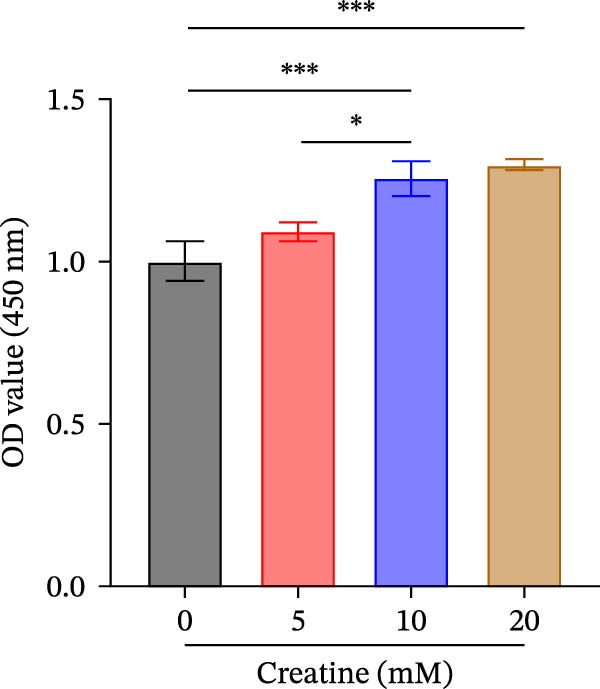
(B)

**Figure:**
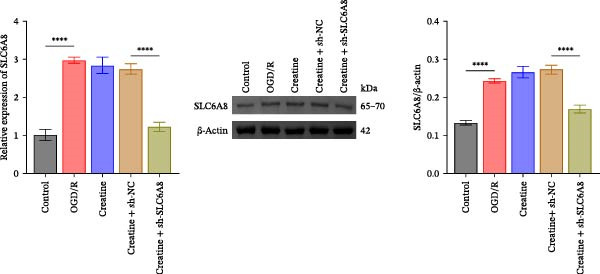
(C)

**Figure:**
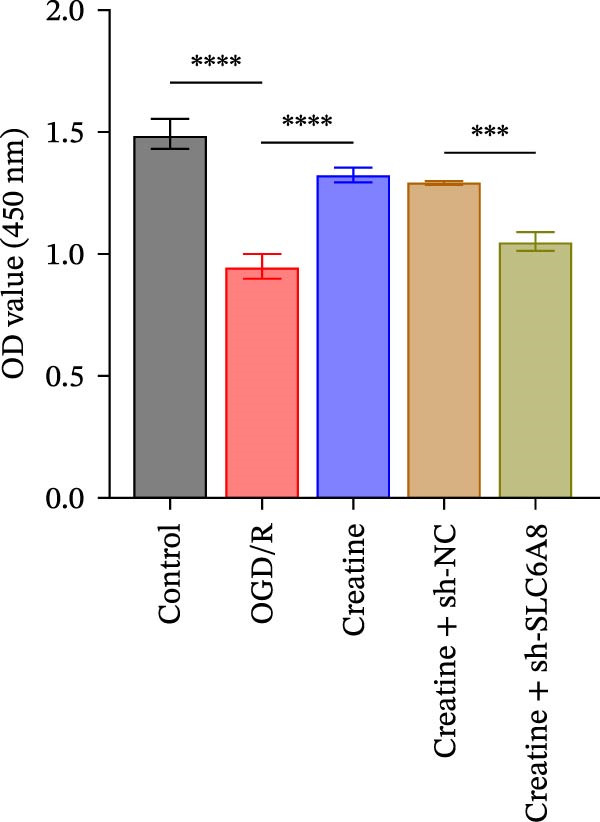
(D)

**Figure:**
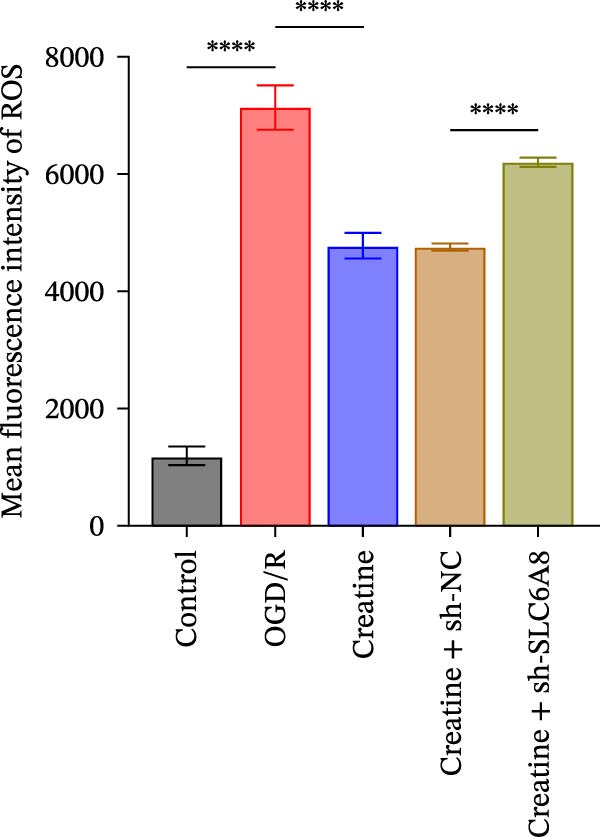
(E)

**Figure:**
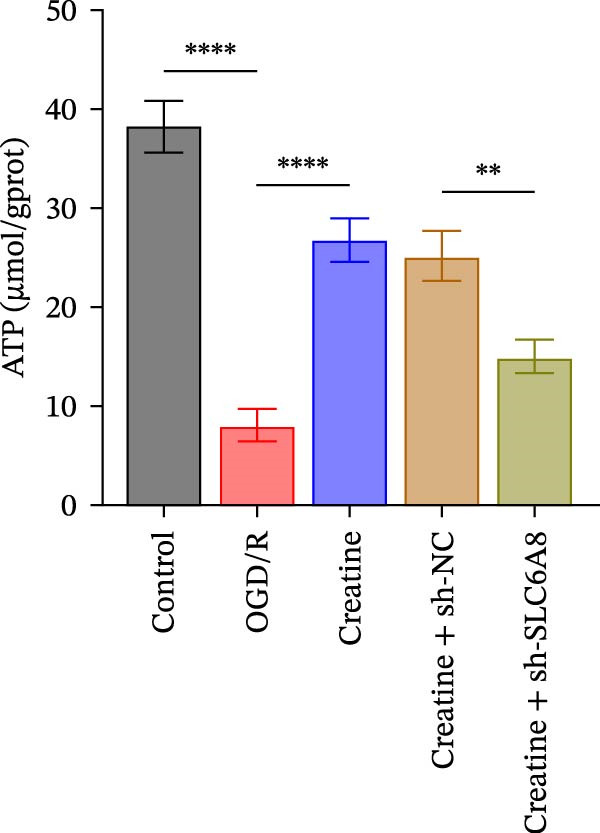
(F)

**Figure:**
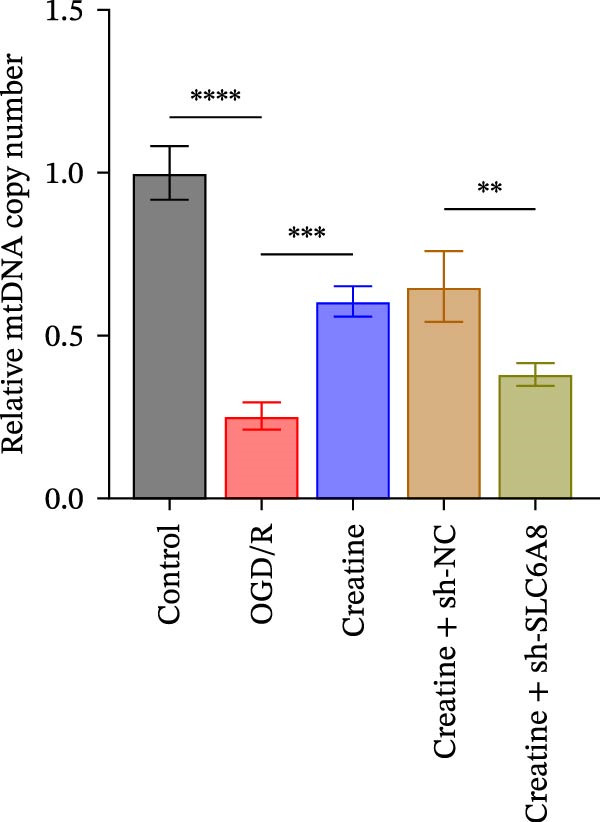
(G)

**Figure:**
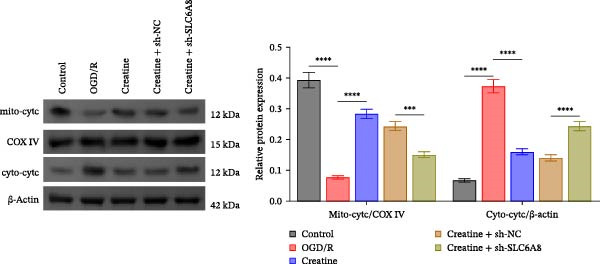
(H)

**Figure:**
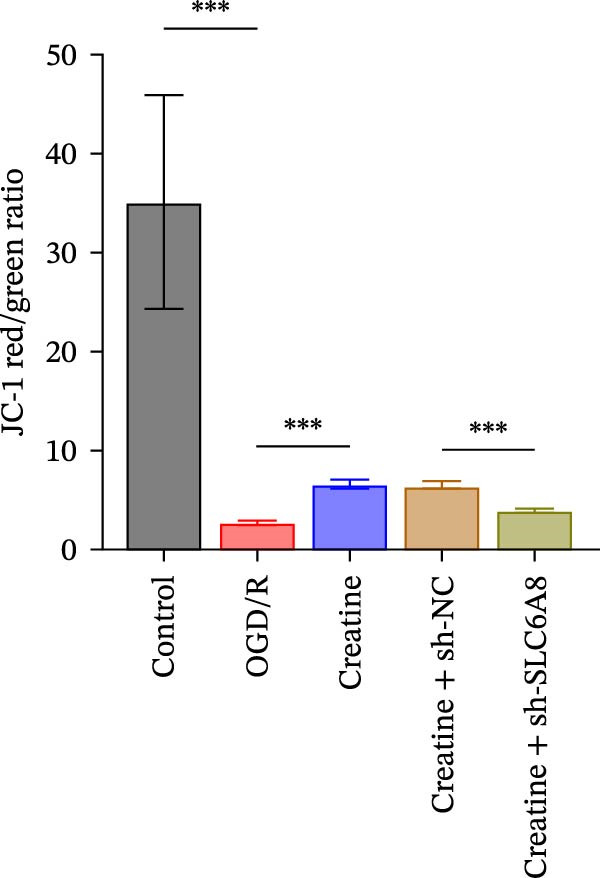
(I)

**Figure:**
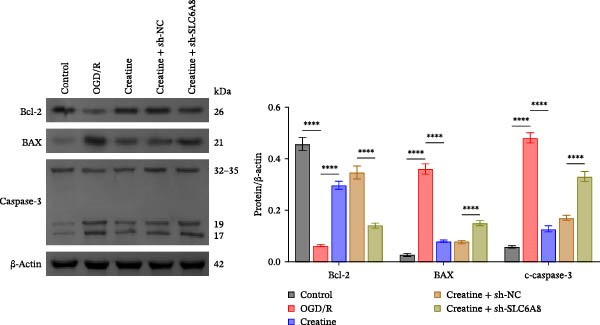
(J)

### 3.5. Verification of Creatine Binding to EARS2 Protein

Creatine has been shown to inhibit ROS production and improve mitochondrial damage [32]. Molecular docking analysis showed that creatine could bind to the EARS2 protein (−5 kcal/mol), mainly by forming stable hydrogen bonds with GLY 52, ARG 55, LEU 277, and LEU 285 residues, and that the compound could interact electrostatically with the GLU249 residue of the protein (Figure 5A). DARTS assay results showed that as the concentration of creatine (0–20 mM) increased, the expression of EARS2 protein became more stable, confirming their binding relationship (Figure 5B). The small molecule pull‐down assay showed that compared with the Input group, 10 mM creatine significantly pulled down the EARS2 protein, proving that creatine can bind to the EARS2 protein (Figure 5C). These findings indicate that the metabolite creatine from RGTL binds directly to the EARS2 protein.

Figure 5The binding of the metabolite creatine with EARS2. (A) Molecular docking analysis of creatine with EARS2. (B) DARTS detection of the binding between creatine and EARS2 in PC12 cells. (C) Small molecule pull‐down assay for detecting creatine‐EARS2 interaction in PC12 cells. *N* = 3 biological repetitions/group.

**Figure:**
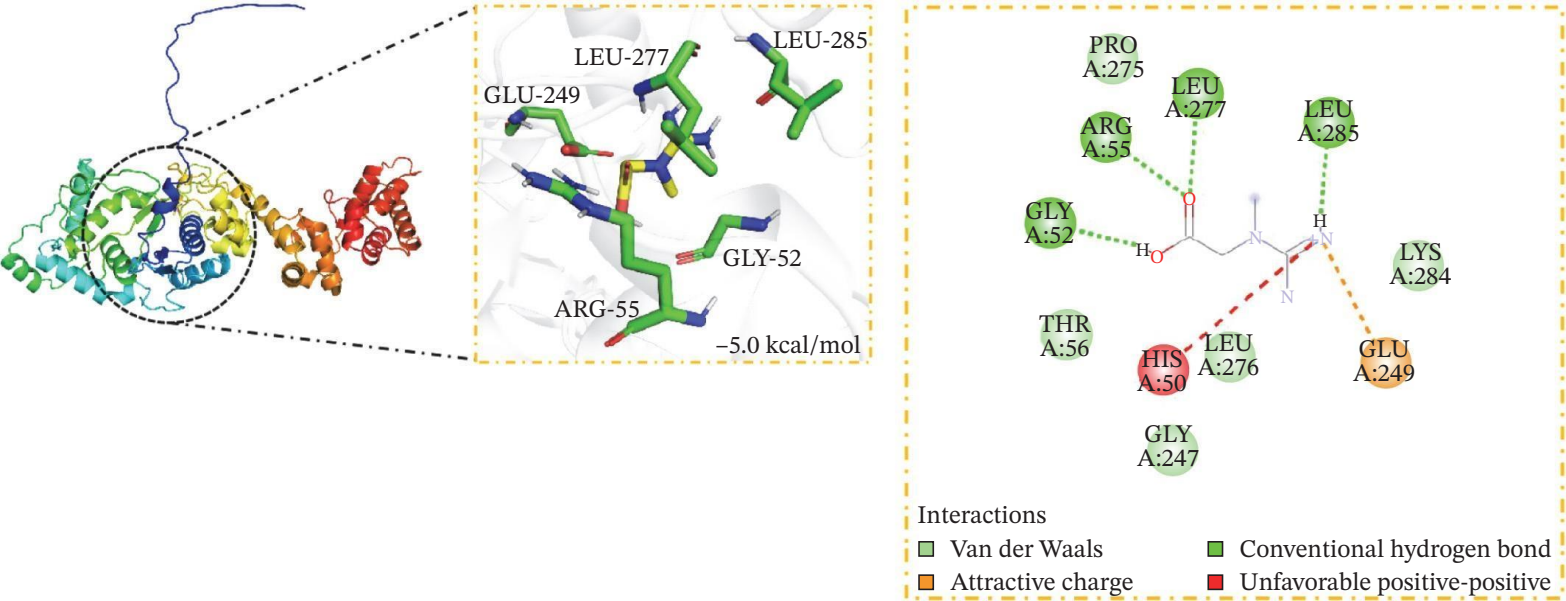
(A)

**Figure:**
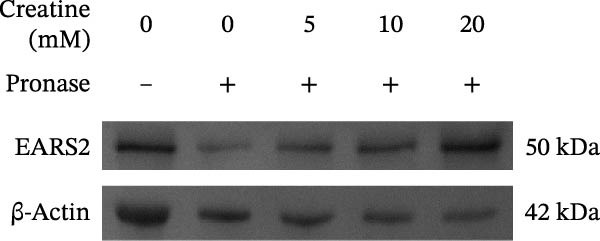
(B)

**Figure:**
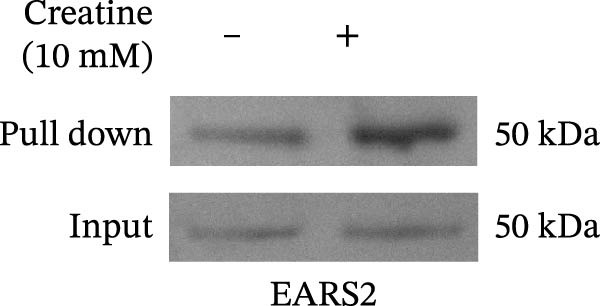
(C)

### 3.6. Creatine Inhibits Mitochondrial Damage and Neuronal Cell Apoptosis by Promoting the Expression of EARS2

To further explore the interaction between creatine and the EARS2 protein, neurons were first transfected with si‐EARS2 for subsequent analysis (Figure 6A). Neurons subjected to OGD/R were then treated with 10 mM creatine. The results showed that EARS2 expression decreased in OGD/R‐induced neurons compared with the Control group (Figure 6B). Creatine treatment promoted EARS2 expression in OGD/R‐induced neurons, while simultaneous intervention with sh‐EARS2 inhibited its expression (Figure 6B). Creatine treatment alleviated the decrease in cell viability in OGD/R‐induced neurons; however, this protective effect was blocked by sh‐EARS2 intervention (Figure 6C). On the basis of creatine treatment, sh‐EARS2 intervention led to increased ROS levels and decreased ATP levels in neurons (Figure 6D, E, Supporting information Figure S1E). Creatine treatment also prevented the reduction in mitochondrial mtDNA copy number, Mito‐Cyt c, and JC‐1 levels, as well as the increase in Cyto‐Cyt c levels in OGD/R‐induced neurons; these effects were abolished following sh‐EARS2 intervention (Figure 6F–H, Supporting information Figure S1F). Transmission electron microscopy revealed that mitochondria in OGD/R cells were circular with irregular and disordered cristae, whereas mitochondria in the creatine‐treated group exhibited linear and parallel cristae. This improvement was inhibited after sh‐EARS2 intervention (Figure 6I). Furthermore, creatine treatment suppressed the decrease in Bcl‐2 expression and the increase in BAX and caspase‐3 expression in OGD/R‐induced neurons, while these effects were reversed by sh‐EARS2 intervention (Figure 6J). These findings confirm that silencing EARS2 blocks the therapeutic effects of creatine on apoptosis and mitochondrial damage in OGD/R‐induced neurons.

Figure 6Creatine alleviates OGD/R‐induced neuronal cell apoptosis and mitochondrial damage through EARS2. (A) WB detection of EARS2 expression to assess transfection efficiency. (B) WB detection of EARS2 expression. (C) CCK8 detection of neuronal cell viability. (D) DCFH‐DA staining to detect ROS levels. (E) ATP content detection using an assay kit. (F) RT‐qPCR detection of mtDNA copy number. (G) WB detection of expression of mitochondrial cytochrome c and cytoplasmic cytochrome c. (H) JC‐1 staining to detect mitochondrial membrane potential. (I) Assessment of mitochondrial damage using transmission electron microscopy (Scale bar = 2.0 μm and 500 nm). (J) WB detection of expression of Bcl‐2, BAX, and caspase‐3. An OGD/R cell model was constructed in vitro with PC12 cells, and then treated with creatine, and transfected with sh‐NC/sh‐EARS2. *N* = 3 biological repetitions/group.

**Figure:**
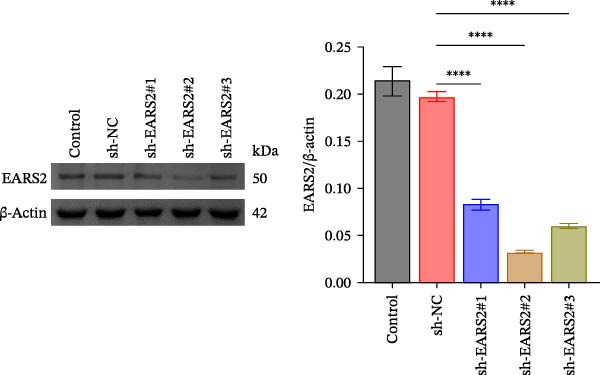
(A)

**Figure:**
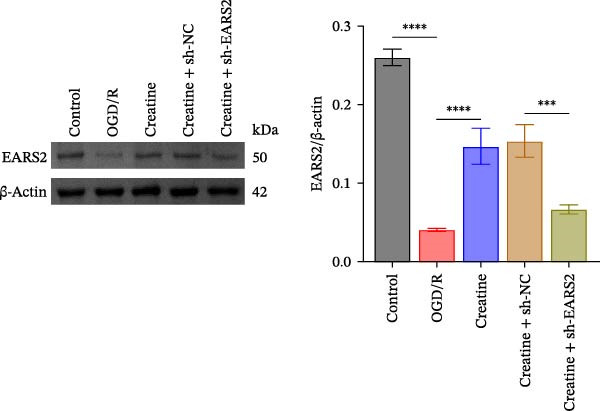
(B)

**Figure:**
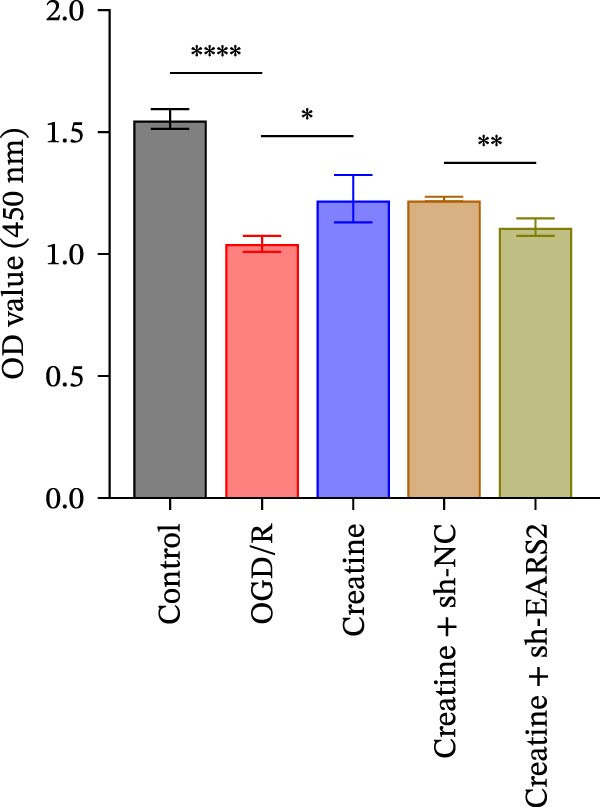
(C)

**Figure:**
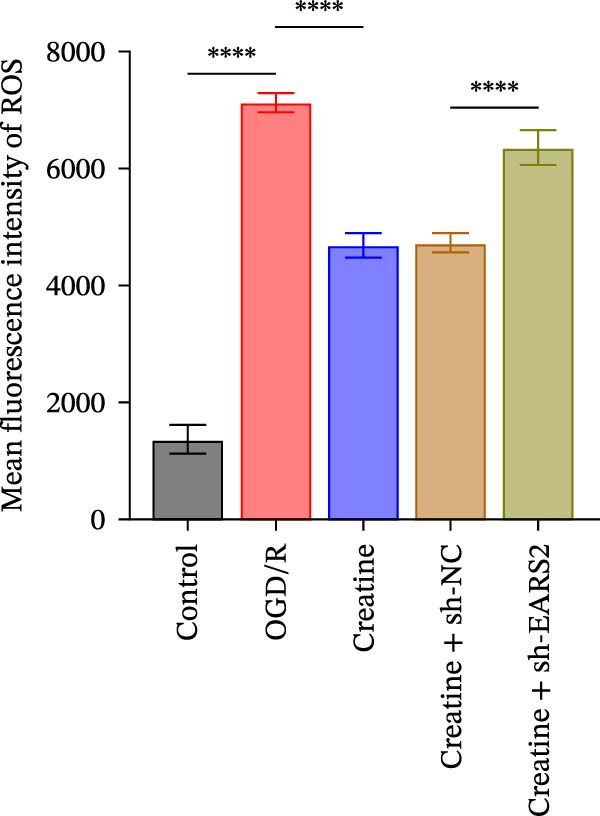
(D)

**Figure:**
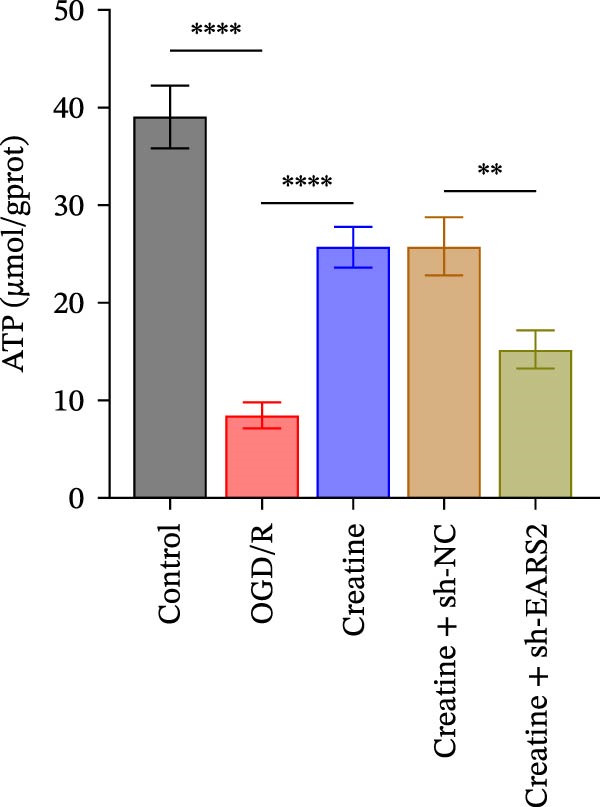
(E)

**Figure:**
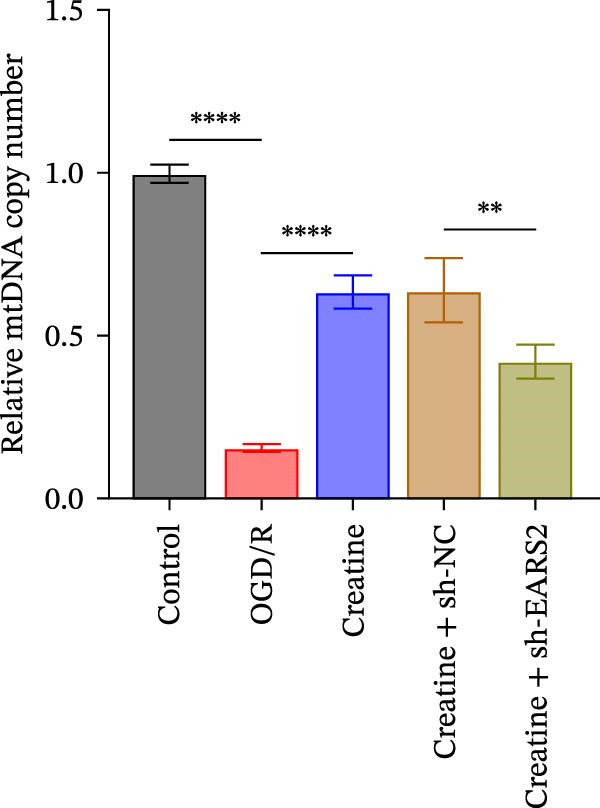
(F)

**Figure:**
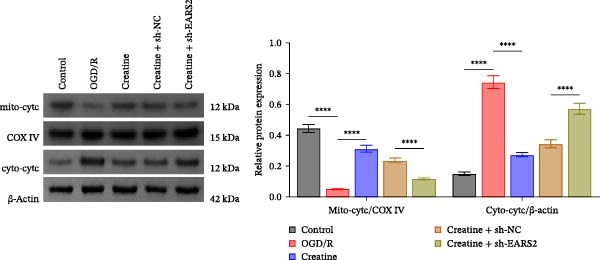
(G)

**Figure:**
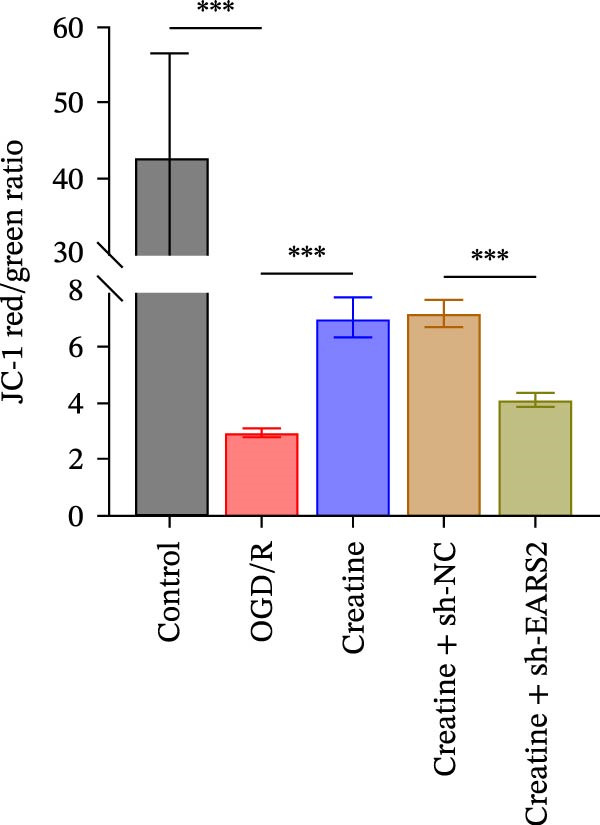
(H)

**Figure:**
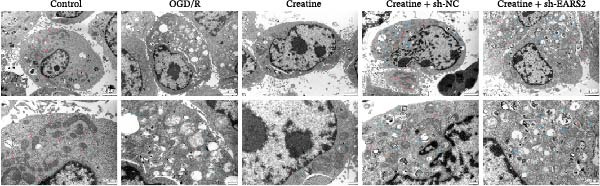
(I)

**Figure:**
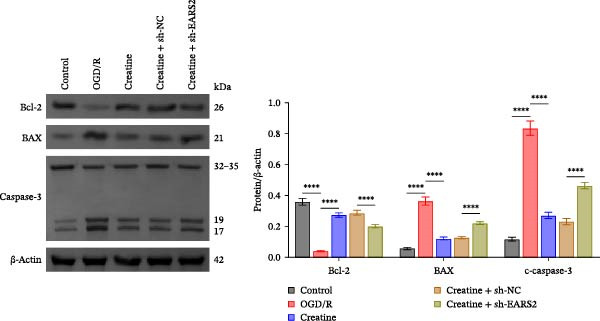
(J)

### 3.7. RGTL Inhibits Mitochondrial Damage and Neuronal Cell Apoptosis by Promoting the Expression of EARS2

Further treatment of neuronal cells with serum containing 5%, 15%, and 20% RGTL decoction showed that all RGTL‐containing serums significantly enhanced cell viability, with the strongest effect observed in the 15% RGTL‐containing serum (Figure 7A). The 15% RGTL‐containing serum upregulated EARS2 protein expression in OGD/R‐induced cells, which was reversed by sh‐EARS2 (Figure 7B). Subsequent treatment with 15% RGTL‐containing serum in OGD/R‐induced cells showed increased cell viability, but this effect was blocked by sh‐EARS2 intervention (Figure 7C). Combined treatment with RGTL‐containing serums and sh‐EARS2 increased ROS levels and decreased ATP levels in neuronal cells (Figure 7D, E, Supporting information Figure S3A). RGTL‐containing serum inhibited the decrease in mtDNA copy number, Mito‐Cyt c, and JC‐1 levels in neuronal cells induced by OGD/R, and increased Cyto‐Cyt c levels; however, this effect was blocked after sh‐EARS2 intervention (Figure 7F–H, Supporting information Figure S3B). Transmission electron microscopy showed that OGD/R‐induced cells had round mitochondria with irregular and disorganized cristae, indicating aggravated damage (Figure 7I). Conversely, treatment with the RGTL‐containing serum alleviated OGD/R‐induced mitochondrial impairment, which was inhibited after sh‐EARS2 intervention (Figure 7I). In addition, RGTL‐containing serums suppressed the reduction of Bcl‐2 expression and the increase in BAX and cleaved caspase‐3 expression in OGD/R‐induced neuronal cells, while these effects were reversed by sh‐EARS2 intervention (Figure 7J). These results confirm that the protective effect of RGTL decoction‐containing serum against OGD/R‐induced apoptosis and mitochondrial damage is mediated through the promotion of EARS2 expression.

Figure 7Silencing EARS2 blocks the therapeutic effects of RGTL‐containing serum on apoptosis and mitochondrial damage induced by OGD/R in neuron cells. (A) CCK8 assay to detect neuron cell viability. (B) WB assay to detect EARS2 expression. (C) CCK8 assay to detect neuron cell viability. (D) DCFH‐DA staining to detect ROS levels. (E) Measurement of ATP levels using a commercial kit. (F) RT‐qPCR assay to detect mtDNA copy number. (G) WB assay to detect expression of mitochondrial cytochrome c and cytoplasmic cytochrome c. (H) JC‐1 staining to detect mitochondrial membrane potential. (I) Transmission electron microscopy to observe mitochondrial damage (Scale bar = 2.0 μm and 500 nm). (J) WB assay to detect expression of Bcl‐2, BAX, and caspase‐3. An OGD/R cell model was constructed in vitro with PC12 cells, then treated with RGTL‐containing serum, and transfected with sh‐NC/sh‐EARS2. *N* = 3 biological repetitions/group.

**Figure:**
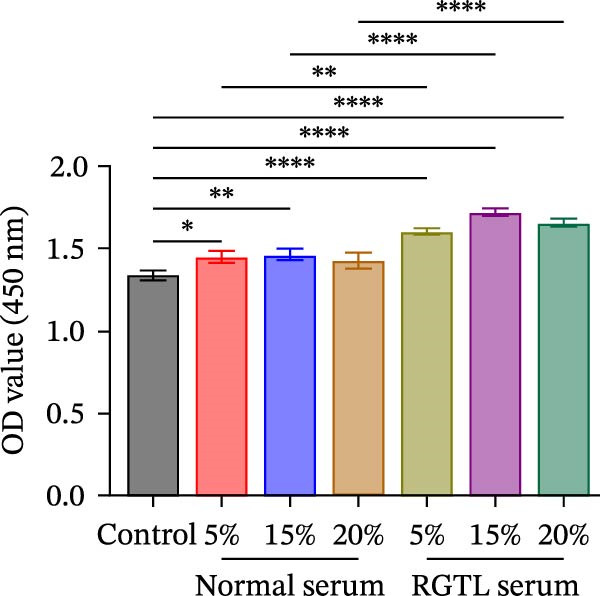
(A)

**Figure:**
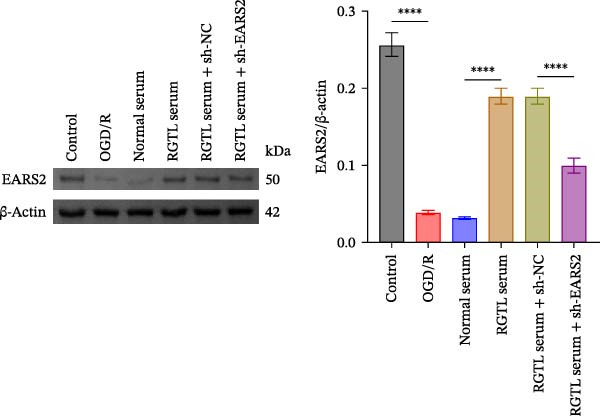
(B)

**Figure:**
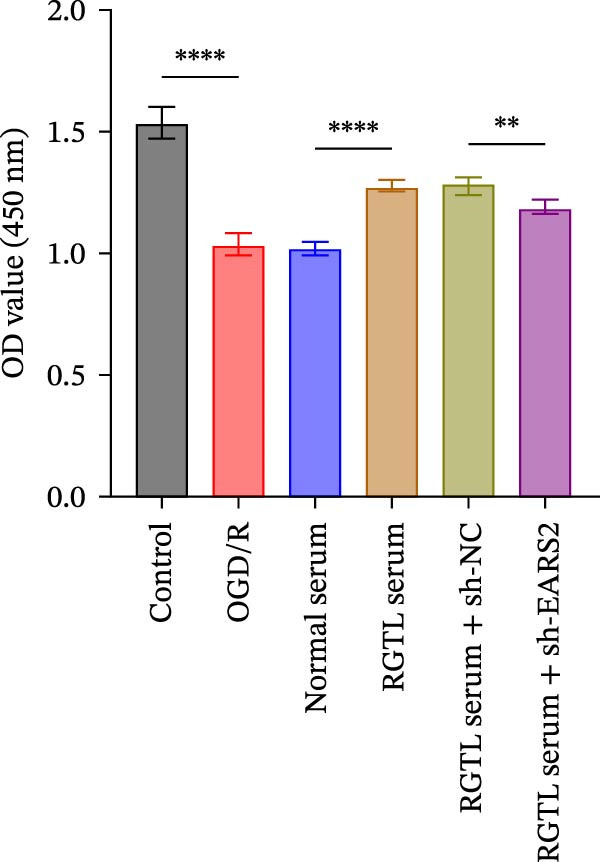
(C)

**Figure:**
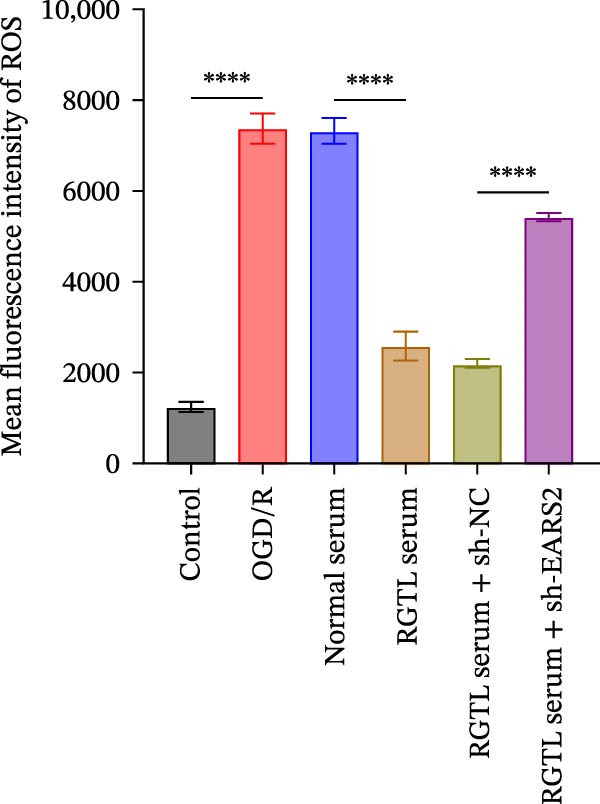
(D)

**Figure:**
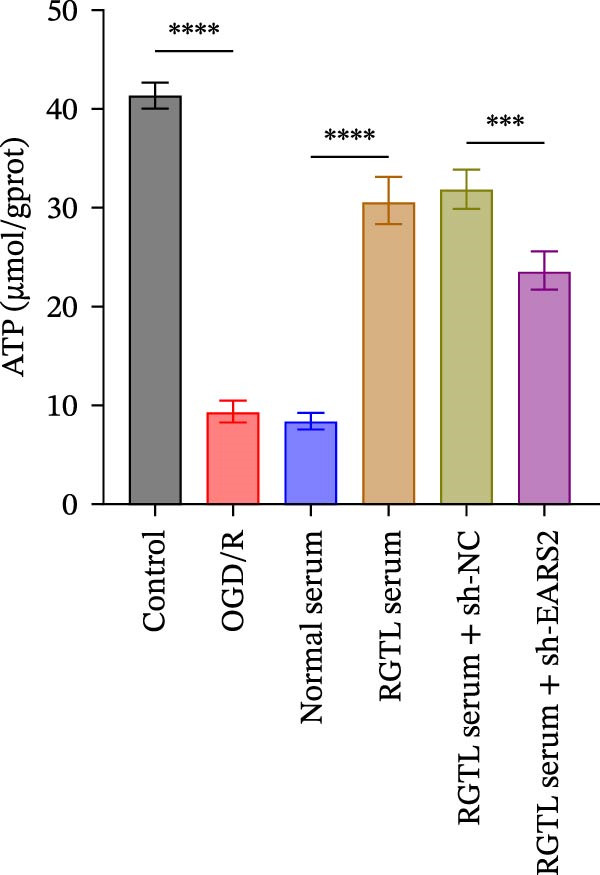
(E)

**Figure:**
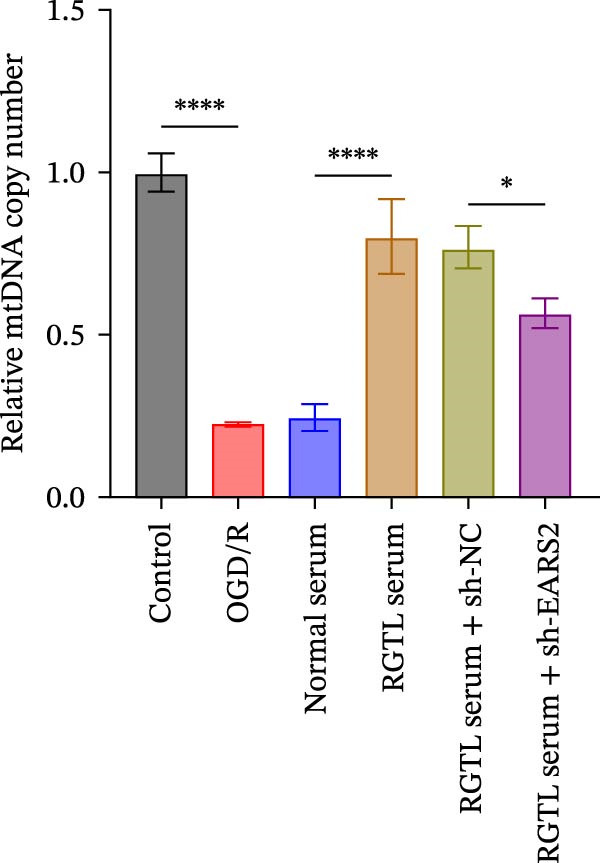
(F)

**Figure:**
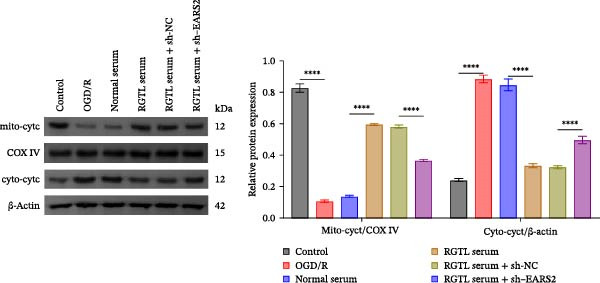
(G)

**Figure:**
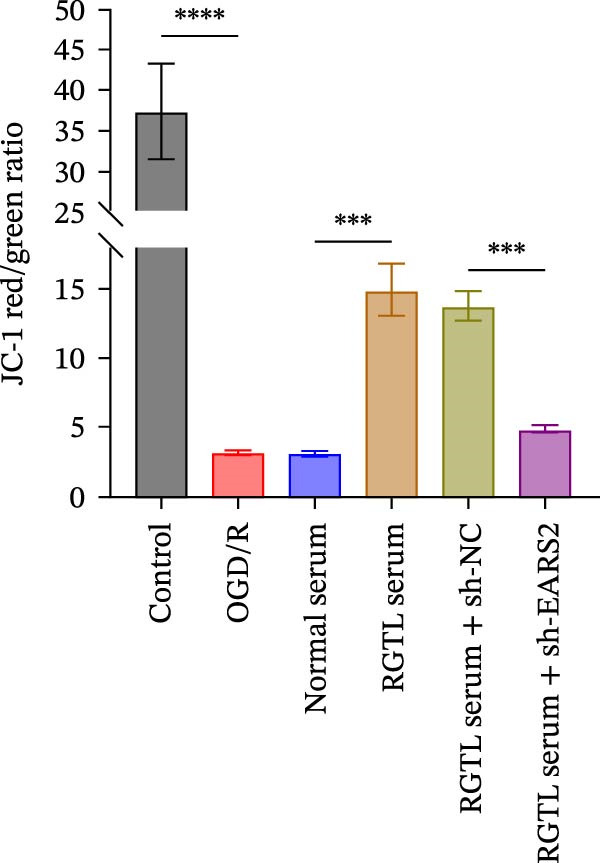
(H)

**Figure:**
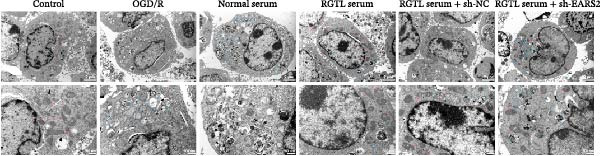
(I)

**Figure:**
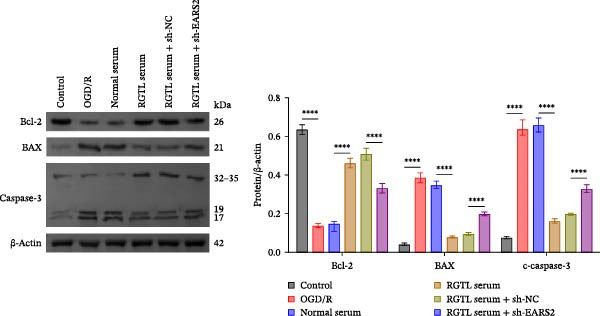
(J)

### 3.8. Creatine Improves Cerebral Ischemic Injury in Rats Through the EARS2 Mitochondrial Pathway

Intervention with creatine reduced the neurological deficit score in MCAO/R rats, while combined intervention with sh‐EARS2 increased it again (Figure 8A). Creatine treatment reduced the infarct area in the brain tissue of MCAO/R rats and alleviated damage to hippocampal tissue and neurons (Figure 8B–D). However, sh‐EARS2 intervention on the basis of creatine treatment increased the infarct area and aggravated hippocampal and neuronal injury (Figure 8B–D). Creatine intervention decreased TNF‐α, IL‐6, and ROS levels in the hippocampal tissue of MCAO/R rats while increasing ATP levels, but these effects were reversed after sh‐EARS2 intervention (Figure 8E–F, Supporting information Figure S3C). Creatine treatment increased the mtDNA copy number, Myto‐Cyt c, and JC‐1 levels in hippocampal tissue while decreasing Cyto‐Cyt c levels (Figure 8G–I, Supporting information Figure S3D). When sh‐EARS2 was introduced, mtDNA copy number, Myto‐Cyt c, and JC‐1 levels decreased, while Cyto‐Cyt c levels increased (Figure 8G–I, Supporting information Figure S3D). Additionally, creatine treatment increased the expression of EARS2 and Bcl‐2 in hippocampal tissue while reducing the expression of BAX and caspase‐3; these effects were reversed following sh‐EARS2 intervention (Figure 8J). Transmission electron microscopy revealed that creatine intervention alleviated mitochondrial damage in the brain tissue of MCAO/R rats, while sh‐EARS2 exacerbated mitochondrial fragmentation and cristae vacuolization (Figure 8K). These results confirm that creatine, a key therapeutic component of RGTL, improves cerebral ischemic injury in rats by regulating the EARS2 mitochondrial pathway.

Figure 8Creatine from RGTL exerts its therapeutic effects by regulating EARS2 to improve cerebral ischemic injury in rats. (A) Assessment of neurological deficit score. (B) TTC staining of cerebral infarction in rat brain tissues. (C) HE staining of brain tissue damage (Scale bar = 100 μm or 25 μm). (D) Nissl staining for hippocampal neuronal injury (Scale bar = 100 μm, or 25 μm). (E) Levels of TNF‐α, IL‐6, and ATP content. (F) Detection of ROS levels. (G) mtDNA copy number. (H) Evaluation of cytochrome c expression in mitochondrial and cytoplasmic compartments. (I) JC‐1 assay to measure mitochondrial membrane potential. (J) WB analysis for assessing the expression of EARS2, Bcl‐2, BAX, and caspase‐3. (K) Transmission electron microscopy showing mitochondrial damage (Scale bar = 2.0 μm and 500 nm). Twenty‐four hours after MCAO/R induction, PC12 cells transfected with sh‐NC or sh‐EARS2 plasmids were transplanted into the hippocampal region of the rats. *N* = 9 mice/group.

**Figure:**
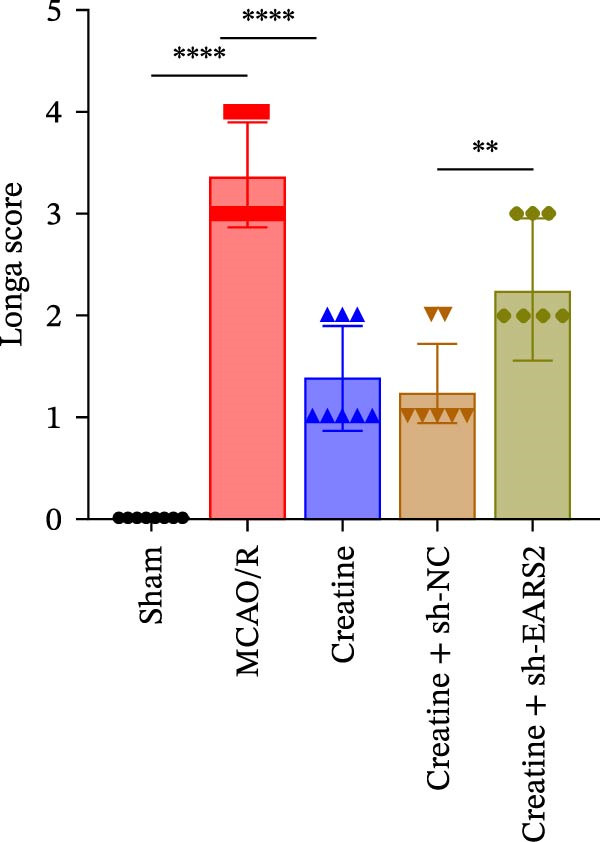
(A)

**Figure:**
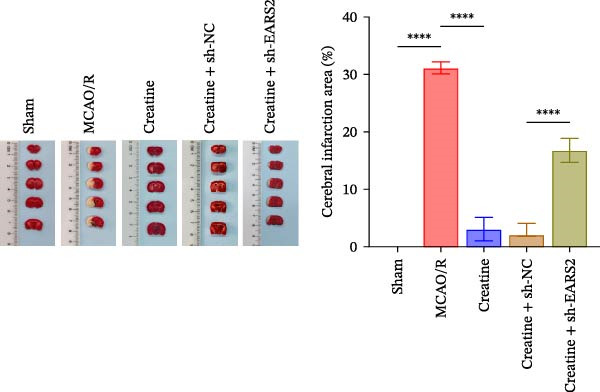
(B)

**Figure:**
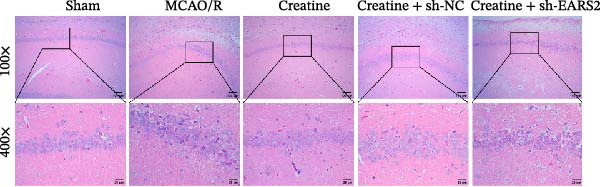
(C)

**Figure:**
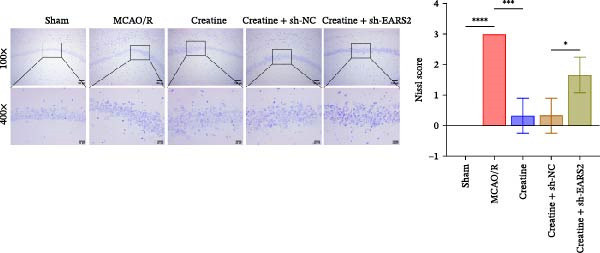
(D)

**Figure:**
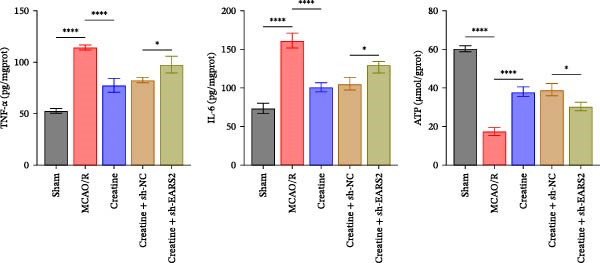
(E)

**Figure:**
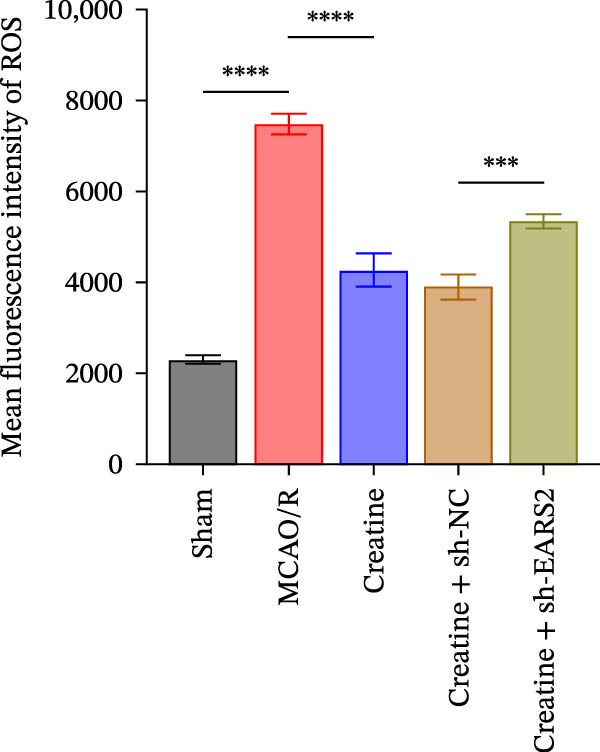
(F)

**Figure:**
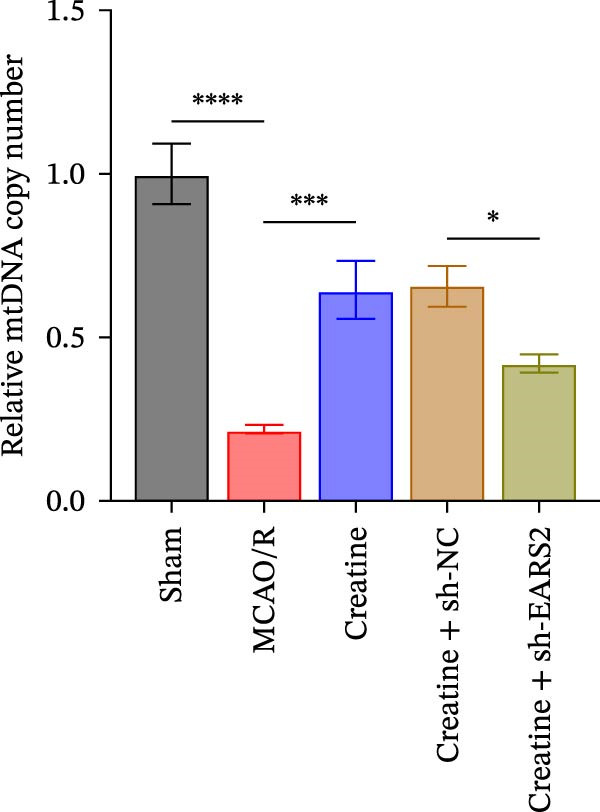
(G)

**Figure:**
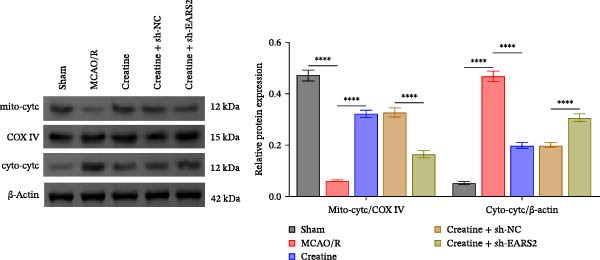
(H)

**Figure:**
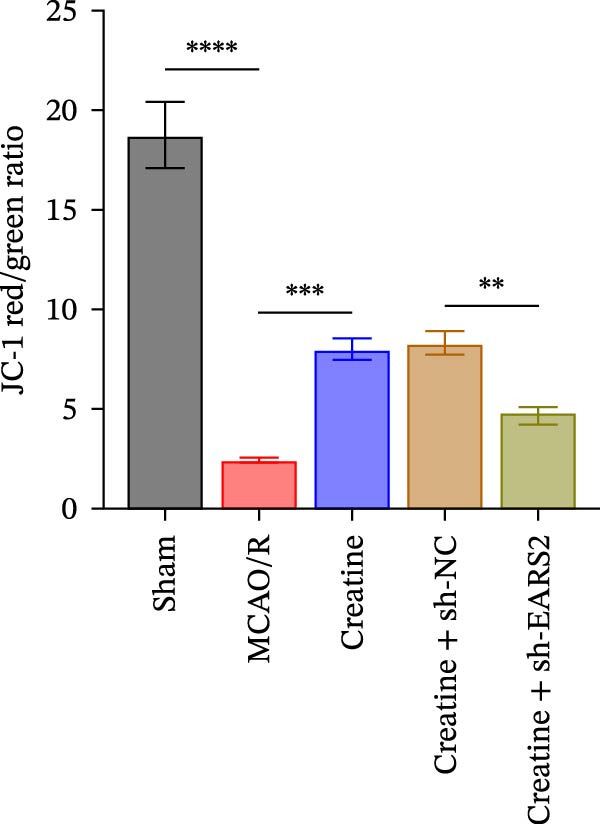
(I)

**Figure:**
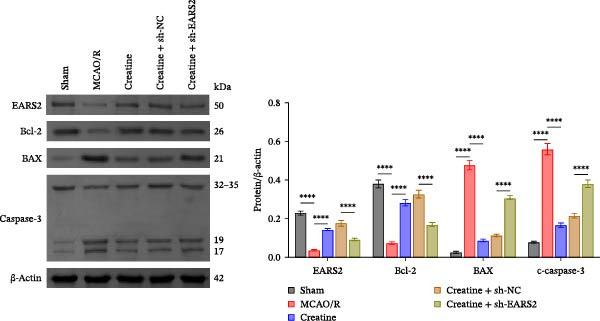
(J)

**Figure:**
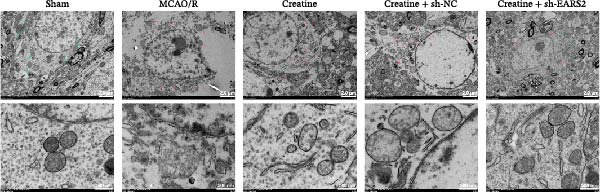
(K)

## 4. Discussion

RGTL consists of Shouwu, Mulberry, Goji Berries, Danshen, Pueraria Root, Angelica, Red Peony, Dilong, and Hawthorn, which were widely used in the clinical treatment of cardiovascular and cerebrovascular diseases. This study is the first to demonstrate that RGTL can promote neurological function recovery after cerebral ischemia using this concept. It is known that oxidative stress and mitochondrial damage are the main mechanisms underlying ischemia‐reperfusion injury in ischemic stroke [40]. Our findings confirm that RGTL treatment reduces TNF‐α, IL‐6, and ROS levels in the hippocampal tissue of MCAO/R rats, increases ATP levels and JC‐1 levels, and alleviates mitochondrial damage, supporting RGTL as an alternative traditional Chinese medicine treatment for cerebral ischemic injury.

Previous studies have explored the targeted molecular mechanisms of active ingredients from Chinese herbal medicines in cerebral ischemia–reperfusion (CIRI) injury. For instance, phelligridimer A, an active compound isolated from the medicinal and edible fungus Sanghuang, may alleviate brain ischemia‐induced damage by restoring mitochondrial function and reducing endoplasmic reticulum stress [41]. Ligustilide, a natural compound extracted from Chuanxiong and Danggui, has been shown to improve mitochondrial function by enhancing mitophagy to alleviate CIRI [42]. Saikosaponin XVII has also been shown to improve mitochondrial metabolic function and inhibit brain ischemic injury [43]. To further investigate the molecular network mechanism of RGTL in CIRI, we combined metabolomics and network pharmacology analyses. Using Venn diagram analysis, we identified the intersection between metabolites and drug components to discover compounds that are not only present in the RGTL decoction but also exhibit significant metabolic alterations following RGTL administration in vivo. Succinic acid and creatine, as active components of RGTL and differential metabolites in hippocampal tissue, may mediate the therapeutic effects of RGTL in MCAO/R rats through MMP3, GAMT, SLC6A8, and CASP3. These findings elucidate the potential pharmacological mechanisms underlying the therapeutic components in RGTL in the treatment of brain ischemic injury.

Our results indicated that RGTL partially reversed and remodeled the metabolic anomalies in the brain tissue of MACO/R rats, which is usually mediated through its anti‐inflammatory and antioxidant stress effects. The heatmap in Figure 2B shows that several metabolites were significantly upregulated or down‐regulated in the model group, while RGTL treatment could reverse these effects, restoring their levels toward those observed in the sham group. This demonstrates RGTL’s restorative effect in promoting metabolic recovery toward normal physiological conditions. However, Figure 2B also reveals that some metabolites did not exhibit such reversal, suggesting that RGTL induces remodeling of certain metabolic pathways during the recovery phase. The identified succinic acid and creatine, both classified as reversed metabolites, suggest that they are the primary active components responsible for RGTL’s therapeutic effects against CIRI. The intersection analysis also confirmed succinic acid and creatine as shared elements, indicating that RGTL not only contains these molecules but also significantly regulates their endogenous levels. Tissue succinate content is known to decrease progressively with increasing graft ischemia time [44]. Treatment with a compound preparation containing succinic acid, pantogam, and chitosan normalized the concentration of downregulated glutathione and activities of glutathione peroxidase, glutathione reductase, and NADPH‐generating enzymes in animals with experimental hypoxia/reperfusion brain injury [45]. Moreover, creatine‐dependent bioenergetics can be enhanced by decreasing ROS through mitochondrial‐targeted peptides [46]. Oral creatine‐modified selenium‐based hyaluronic acid nanogel has also been shown to possess therapeutic potential by restoring mitochondrial biofunction in colitis lesions [47]. This intersection illustrates how RGTL interacts with the endogenous metabolic network of the organism through its chemical components, which bridge “RGTL” and “mitochondrial damage” during the treatment of brain ischemic injury. From hundreds of metabolites and drug components, these findings highlight creatine as the most promising target molecule, which helped enhance the efficiency and specificity of subsequent mechanistic studies.

The creatine kinase system is a major component of the metabolic mechanism, transferring energy between mitochondria and cytosolic solutes [48]. Primary adipocytes exposed to palmitic acid and treated with nitrite exhibit increased mitochondrial respiration, elevated protein expression of total mitochondrial complexes, and upregulation of the gene expression of the creatine transporter protein SLC6A8 [49]. Intracellular creatine enhances antioxidant defense by reducing mitochondrial activity and oxygen consumption, and its accumulation within cells is mediated by SLC6A8 [32]. Our results demonstrate that silencing SLC6A8 blocks the inhibitory effect of creatine on OGD/R‐induced neuronal apoptosis and mitochondrial damage, consistent with previous findings. It has been reported that Kellerin (*Ferula sinkiangensis* K. M. Shen) exerts neuroprotective effects against brain ischemic injury by directly interacting with Akt to inhibit ROS generation in mitochondria [50]. Baicalin improves neuronal PDK2–PDH axis function by suppressing oxidative stress mediated by SDH, which in turn alleviates acute ischemic stroke [51]. Valerianic acid A promotes mitochondrial fusion through the IDO1‐mediated Stat3–Opa1 pathway to improve neuronal function in ischemic stroke [52]. Herein, our study confirms that creatine binds to the EARS2 protein, and cellular and animal experiments showed that silencing EARS2 can block the therapeutic effects of creatine on OGD/R and MCAO/R‐induced neuronal cell apoptosis and mitochondrial damage in rats.

This study investigated the mechanisms via which RGTL decoction and its metabolite creatine may help alleviate ischemic injury and revealed that it could do so by directly regulating energy metabolism within neurons. However, there were several limitations that should be acknowledged. First, we were unable to directly assess the changes in glial cells. Current research reports confirm that maintaining the integrity of the BBB is essential for homeostasis in the CNS. Brain endothelial cells, which structurally constitute the BBB, interact with pericytes, astrocytes, neurons, microglia, and perivascular macrophages in the neurovascular unit (NVU) [53]. Classically activated (M1‐polarized) microglia release pro‐inflammatory mediators and cytotoxic substances to eliminate pathogens. These cells recognize harmful stimuli and secrete inflammatory cytokines such as TNF‐α, IL‐6, and various chemokines, which are important for promoting the M1 activation state [54]. Conversely, alternatively activated (M2‐polarized) microglia exert neuroprotective effects by facilitating tissue repair and regeneration [54]. Our study demonstrated that RGTL exerts pronounced antioxidant and anti‐inflammatory effects, including reductions in ROS, TNF‐α, IL‐6, and other pro‐inflammatory mediators. Previous studies have shown that under normal physiological conditions, astrocytes and microglia maintain CNS homeostasis by providing metabolic and nutritional support to neurons [55]. Necrotic neurons release cytokines such as IL‐6, which induce the activation and aggregation of astrocytes [56–58]. Activated astrocytes induce A1 astrocytes through the secretion of IL‐1α, TNFα, and C1q [59]. A1 astrocytes lose their ability to promote neuronal survival, growth, synaptogenesis, and phagocytosis, while inducing death in neurons and oligodendrocytes. In contrast, A2 astrocytes (healthy astrocytes), which can be directly induced by ischemia, release numerous neurotrophic factors that support neuronal survival and tissue repair, as well as regulate brain homeostasis [58]. Based on our results and existing literature, it can be speculated that RGTL may also exert beneficial indirect effects on glial cells, mainly through its anti‐inflammatory and antioxidant mechanisms, thus, providing an important direction for future research.

In conclusion, we found that RGTL may alleviate mitochondrial dysfunction in neuron cells through the endogenous SLC6A8‐creatine‐EARS2 signaling pathways, thereby exerting neuroprotective effects against brain ischemic injury.

## Author Contributions

**Changze Ou, Zheng-ping Bai, and Guo-heng Hu**: conceived, designed, experiments, data collection, analysis wrote (Changze Ou) edited and commented on the manuscript. **Binbin Chen**: data collection and analysis. **Da-hua Wu and Hua-jun Long**: conceived, designed, planned the study, checked for problems and review.

## Funding

This work was supported by the 2022 National Famous Elderly Chinese Medicine Experts Inheritance Workshop Construction Project (No. [2022] 75), the Natural Science Foundation of Changsha City (kq2208145), the Hunan Provincial Health and Wellness Commission Scientific Research Program Project (C202303078160), and the Natural Science Foundation of Hunan Provincial (No. 2026JJ81855).

## Disclosure

All authors have accepted responsibility for the manuscript’s content and consented to its submission. They have meticulously reviewed all results and unanimously approved the final version of the manuscript.

## Conflicts of Interest

The authors declare no conflicts of interest.

## Supporting Information

Additional supporting information can be found online in the Supporting Information section.

## Supporting information


**Supporting Information 1** Figure S1. The levels of ROS and mitochondrial membrane potential. (A) DCFH‐DA staining to detect ROS levels in rat brain tissues. (B) Flow cytometry to measure mitochondrial membrane potential in rat brain tissues. MCAO/R rats were treated with either RGTL (MCAO/R+RGTL group) or saline (MCAO/R+Vehicle group) to evaluate the therapeutic effect. *N* = 9 mice/group. (C) DCFH‐DA staining to detect ROS levels in PC12 cells. (D) JC‐1 detection of mitochondrial membrane potential in PC12 cells. An OGD/R cell model was constructed in vitro with PC12 cells, and treated with creatine, and transfected with sh‐NC/sh‐SLC6A8. *N* = 3 biological repetitions/group. (E) DCFH‐DA staining to detect ROS levels in PC12 cells. (F) JC‐1 staining to detect mitochondrial membrane potential in PC12 cells. An OGD/R cell model was constructed in vitro with PC12 cells, and then treated with creatine, and transfected with sh‐NC/sh‐EARS2. *N* = 3 biological repetitions/group.

**Supporting Information 2** Figure S2. The neuroprotective effects of RGTL are consistent with the treatment of NAC in MCAO/R mice. (A) Neurological function deficit score. (B) TTC staining detects cerebral infarction in rat brain tissues. (C) HE staining to assess brain tissue damage in rats (Scale bar = 100 μm, or 25 μm). (D) Nissl staining to observe damage in rat hippocampal neurons (Scale bar = 100 μm, or 25 μm). MCAO/R rats were treated with either RGTL (MCAO/R+RGTL group) or NAC (MCAO/R+NAC group) to evaluate the therapeutic effect. *N* = 9 mice/group.

**Supporting Information 3** Figure S3. The detection of ROS and mitochondrial membrane potential levels. (A) DCFH‐DA staining to detect ROS levels in PC12 cells. (B) JC‐1 staining to detect mitochondrial membrane potential in PC12 cells. An OGD/R cell model was constructed in vitro with PC12 cells, then treated with RGTL‐containing serum, and transfected with sh‐NC/sh‐EARS2. *N* = 3 biological repetitions/group. (C) Detection of ROS levels in rat brain tissues. (D) JC‐1 assay to measure mitochondrial membrane potential in rat brain tissues. Twenty‐four hours after MCAO/R induction, PC12 cells transfected with sh‐NC or sh‐EARS2 plasmids were transplanted into the hippocampal region of the rats. *N* = 9 mice/group.

## Data Availability

The datasets used and analyzed during the current study are available from the corresponding author upon reasonable request.
