# Supplementary figures and images for: Rougan Tongluo Decoction Initiates Neuroprotection Against Cerebral Ischemia by Activating the Endogenous SLC6A8‐Creatine‐EARS2 Mitochondrial Pathway
Source: Mediators Inflamm. 2026 Apr 18;2026:4419137. doi: 10.1155/mi/4419137 (PMC13091013; doi:10.1155/mi/4419137)

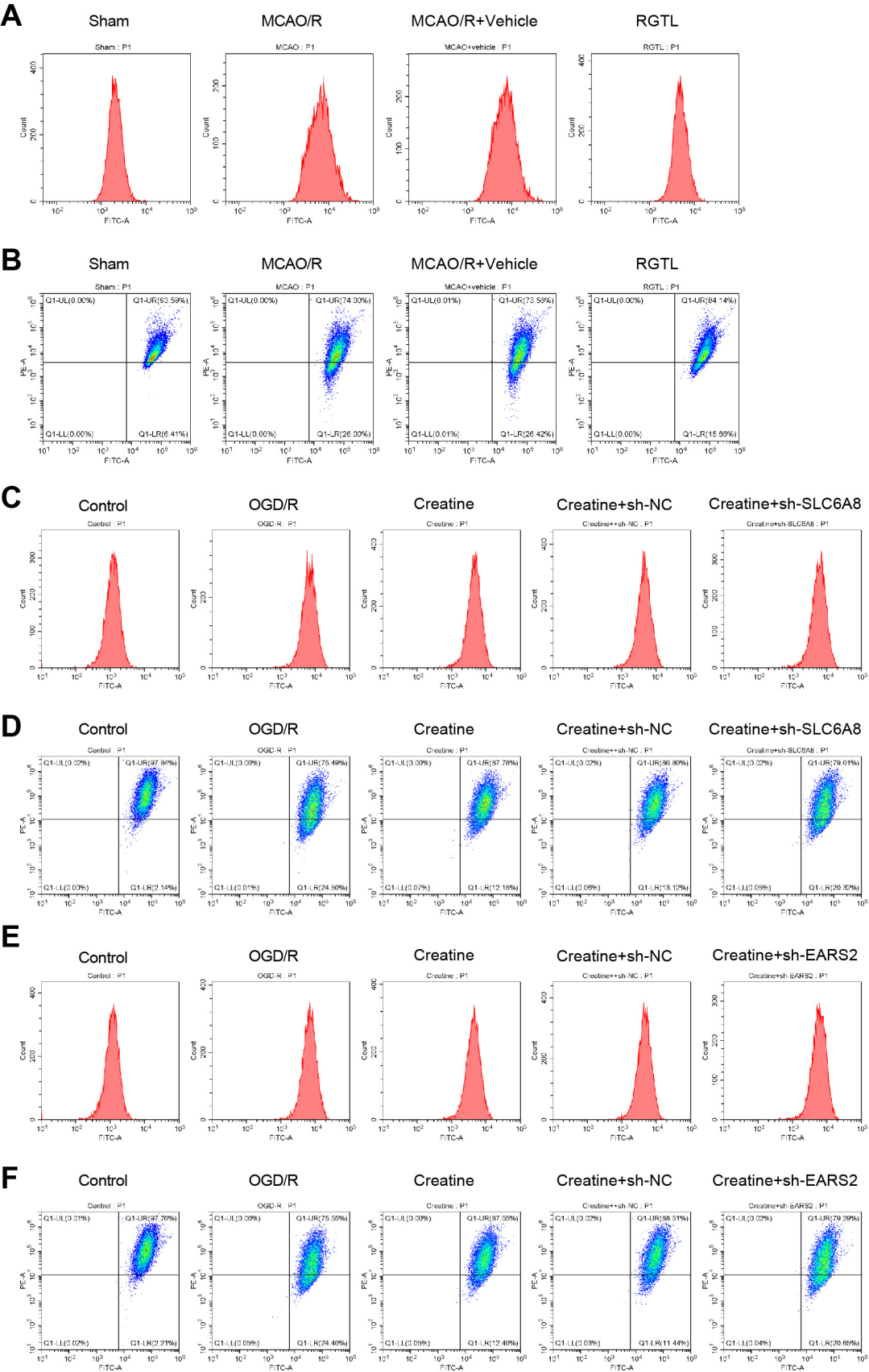

Supplement: Supplementary file 1 — Supporting Information 1 Figure S1. The levels of ROS and mitochondrial membrane potential. (A) DCFH‐DA staining to detect ROS levels in rat brain tissues. (B) Flow cytometry to measure mitochondrial membrane potential in rat brain tissues. MCAO/R rats were treated with either RGTL (MCAO/R+RGTL group) or saline (MCAO/R+Vehicle group) to evaluate the therapeutic effect. N = 9 mice/group. (C) DCFH‐DA staining to detect ROS levels in PC12 cells. (D) JC‐1 detection of mitochondrial membrane potential in PC12 cells. An OGD/R cell model was constructed in vitro with PC12 cells, and treated with creatine, and transfected with sh‐NC/sh‐SLC6A8. N = 3 biological repetitions/group. (E) DCFH‐DA staining to detect ROS levels in PC12 cells. (F) JC‐1 staining to detect mitochondrial membrane potential in PC12 cells. An OGD/R cell model was constructed in vitro with PC12 cells, and then treated with creatine, and transfected with sh‐NC/sh‐EARS2. N = 3 biological repetitions/group. [file MI-2026-4419137-s001.pdf]

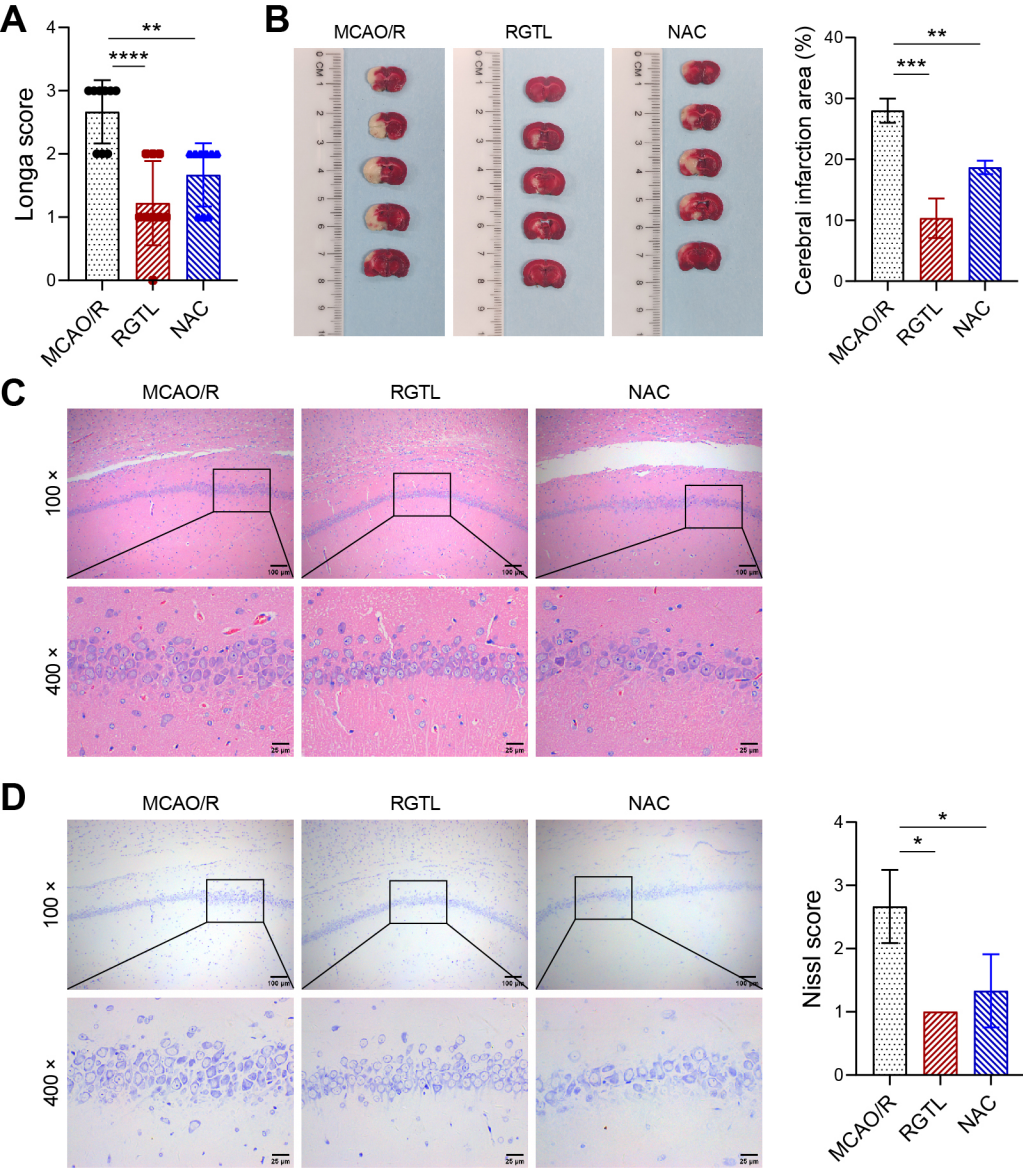

Supplement: Supplementary file 2 — Supporting Information 2 Figure S2. The neuroprotective effects of RGTL are consistent with the treatment of NAC in MCAO/R mice. (A) Neurological function deficit score. (B) TTC staining detects cerebral infarction in rat brain tissues. (C) HE staining to assess brain tissue damage in rats (Scale bar = 100 μm, or 25 μm). (D) Nissl staining to observe damage in rat hippocampal neurons (Scale bar = 100 μm, or 25 μm). MCAO/R rats were treated with either RGTL (MCAO/R+RGTL group) or NAC (MCAO/R+NAC group) to evaluate the therapeutic effect. N = 9 mice/group. [file MI-2026-4419137-s003.pdf]

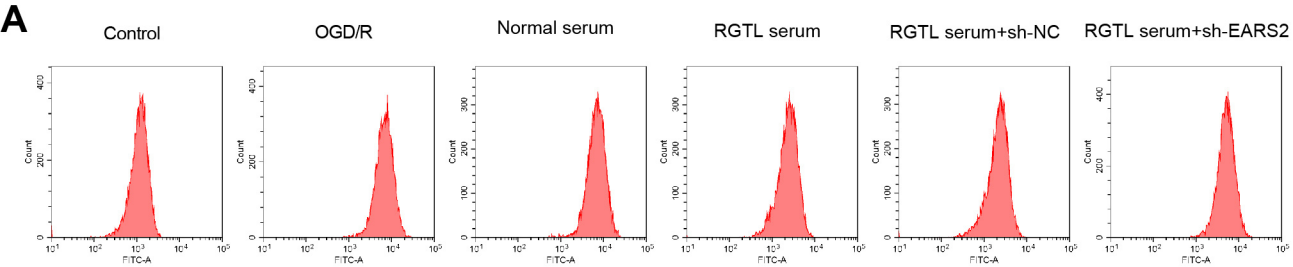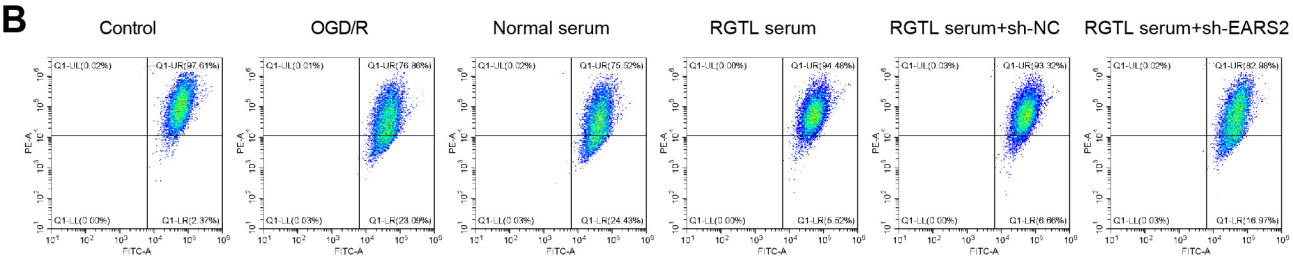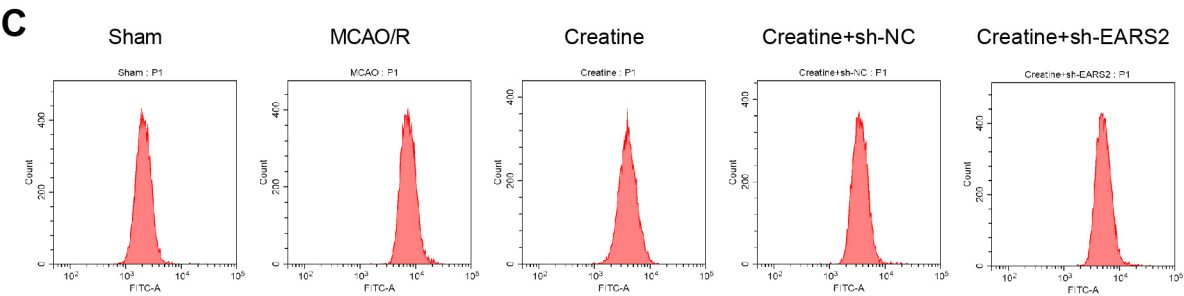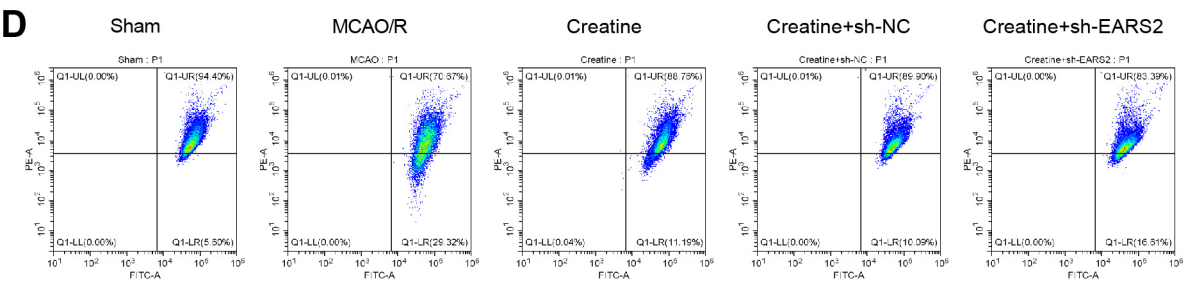

Supplement: Supplementary file 3 — Supporting Information 3 Figure S3. The detection of ROS and mitochondrial membrane potential levels. (A) DCFH‐DA staining to detect ROS levels in PC12 cells. (B) JC‐1 staining to detect mitochondrial membrane potential in PC12 cells. An OGD/R cell model was constructed in vitro with PC12 cells, then treated with RGTL‐containing serum, and transfected with sh‐NC/sh‐EARS2. N = 3 biological repetitions/group. (C) Detection of ROS levels in rat brain tissues. (D) JC‐1 assay to measure mitochondrial membrane potential in rat brain tissues. Twenty‐four hours after MCAO/R induction, PC12 cells transfected with sh‐NC or sh‐EARS2 plasmids were transplanted into the hippocampal region of the rats. N = 9 mice/group. [file MI-2026-4419137-s002.pdf]
